# Spatio-temporal Analysis of the Genetic Diversity of Arctic Rabies Viruses and Their Reservoir Hosts in Greenland

**DOI:** 10.1371/journal.pntd.0004779

**Published:** 2016-07-26

**Authors:** Dennis Hanke, Conrad M. Freuling, Susanne Fischer, Karsten Hueffer, Kris Hundertmark, Susan Nadin-Davis, Denise Marston, Anthony R. Fooks, Anette Bøtner, Thomas C. Mettenleiter, Martin Beer, Thomas B. Rasmussen, Thomas F. Müller, Dirk Höper

**Affiliations:** 1 Friedrich-Loeffler-Institut (FLI), Institute of Diagnostic Virology, Greifswald-Insel Riems, Germany; 2 FLI, Institute of Molecular Virology and Cell Biology, Greifswald-Insel Riems, Germany; 3 FLI, Institute of Epidemiology, Greifswald-Insel Riems, Germany; 4 Department of Veterinary Medicine, University of Alaska, Fairbanks, Alaska, United States of America; 5 Institute of Arctic Biology, University of Alaska, Fairbanks, Alaska, United States of America; 6 Animal Health Microbiology Research, Canadian Food Inspection Agency (CFIA), Centre of Expertise for Rabies, Ottawa Laboratory, Ottawa, Ontario, Canada; 7 Animal and Plant Health Agency (APHA), Wildlife Zoonoses and Vector-borne Diseases Research Group, Addlestone, Surrey, United Kingdom; 8 University of Liverpool, Department of Clinical Infection, Microbiology and Immunology, Liverpool, United Kingdom; 9 DTU National Veterinary Institute, Technical University of Denmark, Lindholm, Kalvehave, Denmark; Centers for Disease Control and Prevention, UNITED STATES

## Abstract

There has been limited knowledge on spatio-temporal epidemiology of zoonotic arctic fox rabies among countries bordering the Arctic, in particular Greenland. Previous molecular epidemiological studies have suggested the occurrence of one particular arctic rabies virus (RABV) lineage (arctic-3), but have been limited by a low number of available samples preventing in-depth high resolution phylogenetic analysis of RABVs at that time. However, an improved knowledge of the evolution, at a molecular level, of the circulating RABVs and a better understanding of the historical perspective of the disease in Greenland is necessary for better direct control measures on the island. These issues have been addressed by investigating the spatio-temporal genetic diversity of arctic RABVs and their reservoir host, the arctic fox, in Greenland using both full and partial genome sequences. Using a unique set of 79 arctic RABV full genome sequences from Greenland, Canada, USA (Alaska) and Russia obtained between 1977 and 2014, a description of the historic context in relation to the genetic diversity of currently circulating RABV in Greenland and neighboring Canadian Northern territories has been provided. The phylogenetic analysis confirmed delineation into four major arctic RABV lineages (arctic 1–4) with viruses from Greenland exclusively grouping into the circumpolar arctic-3 lineage. High resolution analysis enabled distinction of seven geographically distinct subclades (3.I – 3.VII) with two subclades containing viruses from both Greenland and Canada. By combining analysis of full length RABV genome sequences and host derived sequences encoding mitochondrial proteins obtained simultaneously from brain tissues of 49 arctic foxes, the interaction of viruses and their hosts was explored in detail. Such an approach can serve as a blueprint for analysis of infectious disease dynamics and virus-host interdependencies. The results showed a fine-scale spatial population structure in Greenland arctic foxes based on mitochondrial sequences, but provided no evidence for independent isolated evolutionary development of RABV in different arctic fox lineages. These data are invaluable to support future initiatives for arctic fox rabies control and elimination in Greenland.

## Author Summary

Next to dog-mediated rabies, wildlife rabies continues to pose a public health problem, particularly in the northern hemisphere. Control of this zoonosis at the animal source has been proven the most efficient route to reduction of human rabies burden. Successful elimination of red fox-mediated rabies in Western Europe and parts of North America has demonstrated the viability of wildlife rabies control strategies. In some regions, the epidemiology of wildlife rabies is well understood; this is not the case for arctic rabies, particularly in Greenland. Previous molecular epidemiological studies demonstrated the occurrence of one particular arctic rabies virus (RABV) lineage (arctic-3) but were limited by low sample numbers and limited sequence length so as to preclude generation of high resolution phylogenetic analysis. Here, a unique set comprised of 79 complete genome sequences of RABVs from Greenland, Canada, USA (Alaska) and Russia collected over the past four decades was analysed. The use of next generation sequencing (NGS) allowed simultaneous determination of host derived sequences encoding mitochondrial proteins from the same brain tissue of 49 arctic foxes. These sequence data combined with geographical and temporal information permit the study of the genetic diversity and evolution of circulating RABVs in Greenland against the background of reservoir host genetics. The results reveal the existence of a single arctic RABV lineage (arctic-3) in Greenland, which has evolved into multiple distinct variants. These analyses provide an improved knowledge of the evolution of the circulating viruses at the molecular level and a better understanding of the historical perspective of the disease in Greenland compared to other parts of the Arctic. This knowledge will support policy on rabies control in mammalian wildlife reservoirs.

## Introduction

Rabies, an ancient disease known for millennia, is caused by lyssaviruses of the Rhabdoviridae family [1]. The prototypical rabies virus (RABV) has a global distribution and the domestic dog is the host reservoir responsible for the vast majority of the estimated 60,000 human rabies cases annually [2]. Other RABV host reservoirs in terrestrial wildlife are primarily meso-carnivores. In Arctic regions, RABV is believed to be maintained by the arctic fox (*Vulpes lagopus*) [3], which has a circumpolar distribution and has uniquely adapted to the extreme climatic and ecologic conditions of this northern environment [4]. The distribution and group size of arctic foxes are strongly influenced by the distribution and density of prey [5]. Notably, arctic foxes have considerably variable home ranges (5–120 km²) than any other meso-carnivore RABV reservoir host [6–8] and can roam over large areas and migrate over extremely long distances [4,8]. From an epidemiological point of view, this may be an important factor for the spread of RABV in northern Polar regions where rabies-like diseases have been described for about 150 years [9], specifically among sledge dogs in Greenland as early as 1859. However, confirmation of the existence of rabies in Greenland was only provided 100 years later, when Jenkins and Wamberg [10] demonstrated the presence of RABV in dogs and arctic foxes. The disease is considered endemic among the arctic fox population of Greenland [11,12]. A recent epidemiological study of arctic fox rabies in Greenland between 1969 and 2011 revealed that the disease flared up every 5–10 years on average, whereby most rabid foxes were reported from southern Greenland [13].

Historically, some properties of arctic RABVs were regarded as “atypical” [14]. However, early genetic virus characterizations based on the nucleoprotein (N) gene clearly identified it as RABV but as a separate virus lineage designated as “arctic” [15]. This lineage circulates throughout the circumpolar region including northern regions of North America, Europe, and Asia. More detailed phylogenetic analyses revealed that the arctic RABV variant can be further delineated into at least four distinct groups [16–18]. The arctic-1 lineage, recovered from southern Ontario, Canada, in the late 20th and early 21st centuries, represented the remnants of an epidemic that spread from northern Canada in the mid-1900s; reports of this strain are now rare due to the rabies control program carried out by provincial authorities. The arctic-4 lineage has only ever been recovered from regions of Alaska and viruses of the arctic-2 lineage appear to be restricted to Siberia, the Russian Far East, and Alaska. In contrast, lineage arctic-3 has a circumpolar distribution [17]. Arctic rabies was also detected on the European Svalbard Islands with the prevailing RABV lineage having a closer phylogenetic relationship to those occurring in the polar regions of Russia [19]. Viruses closely related to those of the arctic clade have been designated as ‘arctic-like’ or ‘arctic-related’ but have a broad distribution in central, east, and southeast Asia [18,20].

Published phylogenetic analysis demonstrated that RABV isolates from Greenland belong to the arctic-3 lineage [16,17]. These studies did not allow for a more comprehensive evolutionary analysis as the datasets were restricted in terms of the number of samples, time, geographic origin, and sequence length. In this present study, a comprehensive panel of 58 RABVs from Greenland between 1990 and 2014 was analyzed. Additionally, 24 arctic RABVs from Canada/Alaska and Russia were also sequenced and added to this dataset to provide some context to the situation in Greenland. The principal objectives were (i) to infer the viral phylogenetic relationships in space and time based on complete genome sequences and (ii) to gain more insights into the contribution of the host population to the spatial spread of individual RABVs in Greenland. In particular, evidence based on sequence analysis for any links between phylogenetic clusters of arctic RABV and the arctic fox population in Greenland was sought.

## Materials and Methods

### Origin of viruses

All samples in this study were either taken from officially implemented passive rabies surveillance programs or already existing collections at the (i) DTU National Veterinary Institute, Technical University of Denmark, Denmark, (ii) Canadian Food Inspection Agency (CFIA), Canada, (iii) Friedrich-Loeffler-Institut (FLI), Germany, and (iv) Animal and Plant Health Agency (APHA), UK. Samples (Table 1) comprised original clinical brain samples submitted for passive surveillance from arctic foxes (*Vulpes lagopus*), red foxes (*Vulpes vulpes*), dogs (*Canis lupus familiaris*), cats (*Felis silvestris catus*), sheep (*Ovis aries*), and a long-tailed ground squirrel (*Citellus undulates*) collected between 1977 and 2014 in Greenland, Northern Canada, Alaska and Russia that tested positive in the direct fluorescent antibody test (FAT, [21]). Because this is a multi-center study, RNA extraction, library preparation and sequencing were done using slightly different protocols. The 3 protocols are briefly outlined in the following paragraphs and the respective protocol is denoted for each sample in Table 1. In no case was 5’- or 3’-RACE performed to confirm the genome termini. The accessions for all RABV genome sequences generated in this study are given in Table 1.

**Table 1:** Details of RABV samples investigated in this study.

Sample name for this study^1^	Year	Month^1^	Host	Country	Location^1^	Area code^1^	Gauss-Krueger coordinates^1^		Arctic fox mitochondrial sequences available ^1^^,^^2^	RABV INSDC accession^2^	Sequencing Protocol
							N	W			
Gra21.05-GRL-1-AF-2005	2005	12	Arctic fox	Greenland	Siorapaluk/Qaanaq	1	77.47	70.46	yes	LM645017	1
Gra07.06-GRL-1-AF-2006	2006	3	Arctic fox	Greenland	Savissivik	1	76.01	65.08	yes	LM645015	1
Gra14.06-GRL-1-AF-2006	2006	4	Arctic fox	Greenland	Pituffik	1	76.32	68.45	yes	LM645016	1
Gra03.13-GRL-1-AF-2013	2013	3	Arctic fox	Greenland	Qaasuitsup/Thule	1	76.53	68.70	yes	LT598541	1
Gra23.06-GRL-2-AF-2006	2006	11	Arctic fox	Greenland	Torsukattak/Qeqertaq	2	70.03	51.27	yes	LM645018	1
Gra03.10-GRL-2-AF-2010	2010	6	Arctic fox	Greenland	Nuussuaq/Upernavik	2	74.07	57.04	yes	LM645019	1
Gra01.13-GRL-2-AF-2013	2013	3	Arctic fox	Greenland	Upernavik/Kullorsuaq	2	74.58	57.22	yes	LT598539	1
Gra02.13-GRL-2-AF-2013	2013	3	Arctic fox	Greenland	Upernavik/Aappilattoq	2	60.13	44.30	yes	LT598538	1
Gra03.06-GRL-3-AF-2006	2006	2	Arctic fox	Greenland	Qasigiannguit	3	68.49	51.05	yes	LM645025	1
Gra04.06-GRL-3-AF-2006	2006	2	Arctic fox	Greenland	Niaqornaarsuk	3	68.18	53.27	yes	LM645021	1
Gra05.06-GRL-3-AF-2006	2006	2	Arctic fox	Greenland	Aasiaat	3	68.49	51.05	yes	LM645026	1
Gra08.06-GRL-3-AF-2006	2006	3	Arctic fox	Greenland	Aasiaat	3	68.42	52.52	yes	LM645023	1
Gra09.06-GRL-3-AF-2006	2006	3	Arctic fox	Greenland	Iginniarfik	3	68.85	53.10	yes	LM645028	1
Gra10.06-GRL-3-AF-2006	2006	3	Arctic fox	Greenland	Aasiaat	3	68.42	52.52	yes	LM645024	1
Gra24.06-GRL-3-D-2006	2006	11	Dog	Greenland	Kangaatsiaq	3	68.18	53.27	ND	LM645022	1
Gra25.06-GRL-3-AF-2006	2006	11	Arctic fox	Greenland	Qasigiannguit	3	68.49	51.05	yes	LM645027	1
Gra02.07-GRL-3-AF-2007	2007	1	Arctic fox	Greenland	Kangaatsiaq	3	68.18	53.27	yes	LM645020	1
Gra01.14-GRL-3-AF-2014	2014	10	Arctic fox	Greenland	Kangaatsiaq/Ikerasaarsuk	3	68.14	53.44	yes	LT598543	1
Gra16.06-GRL-4-AF-2006	2006	5	Arctic fox	Greenland	Kangerlussuaq	4	67.04	50.41	yes	LM645031	1
Gra09.07-GRL-4-AF-2007	2007	4	Arctic fox	Greenland	Sisimiut	4	66.55	53.40	yes	LM645029	1
Gra10.07-GRL-4-AF-2007	2007	5	Arctic fox	Greenland	Sisimiut	4	66.55	53.40	yes	LM645030	1
Gra02.14-GRL-4-AF-2014	2014	9	Arctic fox	Greenland	Kangerlussuaq	4	67.01	50.70	yes	LT598540	1
Gra03.14-GRL-4-AF-2014	2014	10	Arctic fox	Greenland	Kangerlussuaq	4	67.01	50.70	yes	LT598542	1
Gra02.10-GRL-5-AF-2010	2010	5	Arctic fox	Greenland	Qoqqut/Nuuk	5	64.16	50.54	yes	LM645033	1
Gra05.10-GRL-5-AF-2010	2010	6	Arctic fox	Greenland	Kobbefjord/Nuuk	5	64.10	51.44	yes	LM645032	1
Gra10.08-GRL-6-AF-2008	2008	11	Arctic fox	Greenland	Paamiut	6	62.00	49.43	yes	LM645037	1
Gra07.09-GRL-6-AF-2009	2009	5	Arctic fox	Greenland	Godthåbsfjorden/Nuuk	6	60.55	48.15	yes	LM645034	1
Gra13.09-GRL-6-AF-2009	2009	12	Arctic fox	Greenland	Paamiut	6	62.00	49.43	yes	LM645038	1
Gra06.10-GRL-6-AF-2010	2010	9	Arctic fox	Greenland	Paamiut	6	62.00	49.43	yes	LM645036	1
Gra07.10-GRL-6-AF-2010	2010	9	Arctic fox	Greenland	Kangilinnguit/Ivittuut	6	61.14	48.60	yes	LM645035	1
Gra18.05-GRL-7-AF-2005	2005	11	Arctic fox	Greenland	Qaqortoq	7	60.43	46.03	yes	LM645048	1
Gra01.06-GRL-7-AF-2006	2006	1	Arctic fox	Greenland	Narsaq	7	60.54	46.03	yes	LM645051	1
Gra02.06-GRL-7-AF-2006	2006	2	Arctic fox	Greenland	Narsaq	7	60.54	46.03	yes	LM645052	1
Gra06.06-GRL-7-C-2006	2006	3	Cat	Greenland	Qaqortoq	7	60.43	46.03	ND	LM645046	1
Gra20.06-GRL-7-AF-2006	2006	9	Arctic fox	Greenland	Qaqortoq	7	60.43	46.03	no	NA	1
Gra21.06-GRL-7-AF-2006	2006	9	Arctic fox	Greenland	Narsarsuaq	7	61.08	45.26	yes	LM645055	1
Gra26.06-GRL-7-AF-2006	2006	11	Arctic fox	Greenland	Qaqortoq	7	60.43	46.03	no	NA	1
Gra04.07-GRL-7-AF-2007	2007	1	Arctic fox	Greenland	Igaliko	7	60.59	45.25	yes	LM645053	1
Gra05.07-GRL-7-AF-2007	2007	2	Arctic fox	Greenland	Qaqortoq	7	60.43	46.03	yes	LM645045	1
Gra15.07-GRL-7-S-2007	2007	10	Sheep	Greenland	Narsarsuaq	7	61.08	45.26	ND	LM645054	1
Gra3a.07-GRL-7-AF-2007	2007	1	Arctic fox	Greenland	Qaqortoq	7	60.43	46.03	yes	LM645049	1
Gra3b.07-GRL-7-AF-2007	2007	1	Arctic fox	Greenland	Qaqortoq	7	60.43	46.03	yes	LM645050	1
Gra02.08-GRL-7-AF-2008	2008	4	Arctic fox	Greenland	Ammassalik	7	65.43	37.35	yes	LM645056	1
Gra03.08-GRL-7-AF-2008	2008	4	Arctic fox	Greenland	Nanortalik	7	60.08	45.14	yes	LM645041	1
Gra13.08-GRL-7-AF-2008	2008	12	Arctic fox	Greenland	Qaqortoq	7	60.43	46.03	yes	LM645047	1
Gra01.09-GRL-7-S-2009	2009	1	Sheep	Greenland	Nanortalik	7	60.08	45.14	ND	LM645039	1
Gra02.09-GRL-7-S-2009	2009	1	Sheep	Greenland	Nanortalik	7	60.08	45.14	ND	LM645040	1
Gra03.09-GRL-7-AF-2009	2009	3	Arctic fox	Greenland	Nanortalik	7	60.08	45.14	yes	LM645042	1
Gra04.09-GRL-7-AF-2009	2009	4	Arctic fox	Greenland	Qaqortoq	7	60.43	46.03	yes	NA	1
Gra01.10-GRL-7-AF-2010	2010	1	Arctic fox	Greenland	Qaqortoq	7	60.43	46.03	yes	LM645043	1
Gra01.11-GRL-7-AF-2011	2011	3	Sheep	Greenland	Qaqortoq	7	60.43	46.03	ND	LM645044	1
13232-CAN-NT-AF-1977	1977	-	Arctic fox	Canada	Northwest Territories	NT	NA	NA	yes	LT598537	1
RV1391-GRL-1-F-1990	1990	3	Arctic fox	Greenland	Thule	1	NA	NA	no	KX036361	3
RV1415-GRL-4-F-2001	2001	12	Arctic fox	Greenland	Kangerlussuaq	4	NA	NA	no	KX036362	3
RV1416-GRL-5-AF-2002	2002	1	Arctic fox	Greenland	Nuuk	5	NA	NA	yes	KX036363	3
RV1417-GRL-3-AF-2002	2002	1	Arctic fox	Greenland	Kangaasiaq	3	NA	NA	yes	KX036364	3
RV1418-GRL-4-AF-2002	2002	1	Arctic fox	Greenland	Kangerlussuaq	4	NA	NA	yes	KX036365	3
RV1419-GRL-3-AF-2002	2002	10	Arctic fox	Greenland	Ilulissat	3	NA	NA	yes	KX036366	3
RV1420-GRL-4-AF-2002	2002	10	Arctic fox	Greenland	Kangerlussuaq	4	NA	NA	yes	KX036367	3
07V1483RFX-USA-AK-RF-2007	2007	NA	Red fox	USA	NA	AK	NA	NA	ND	KU198471	2
89V809AFX-USA-AK-AF-1989	1989	NA	Arctic fox	USA	Barrow	AK	NA	NA	no	KU198460	2
90V814AFX-USA-AK-AF-1990	1990	NA	Arctic fox	USA	Kwigillingok	AK	NA	NA	no	KU198462	2
90V820RFX-USA-AK-RF-1990	1990	NA	Red fox	USA	Cold Bay	AK	NA	NA	ND	KU198463	2
12N0215RFX-CAN-NL-RF-2012	2012	NA	Red fox	Canada	St. John's Co	NL	NA	NA	ND	KU198473	2
12N0280RFX-CAN-NL-RF-2012	2012	NA	Red fox	Canada	St. John's Co	NL	NA	NA	ND	KU198474	2
14N0601RFX-CAN-NL-RF-2014	2014	NA	Red fox	Canada	Nain	NL	NA	NA	ND	KU198479	2
12L1020AFX-CAN-NT-AF-2012	2012	NA	Arctic fox	Canada	Holman	NT	NA	NA	no	KU198472	2
13L0040AFX-CAN-NT-AF-2013	2013	NA	Arctic fox	Canada	Holman	NT	NA	NA	no	KU198475	2
13L0094AFX-CAN-NT-AF-2013	2013	NA	Arctic fox	Canada	Paulatuk	NT	NA	NA	no	KU198476	2
90L2968AFX-CAN-NT-AF-1990	1990	NA	Arctic fox	Canada	Grise Fiord	NT	NA	NA	no	KU198461	2
91L0085AFX-CAN-NT-AF-1991	1991	NA	Arctic fox	Canada	Cambridge Bay	NT	NA	NA	no	KU198464	2
93N1395AFX-CAN-NT-AF-1993	1993	NA	Arctic fox	Canada	Resolute Bay	NT	NA	NA	no	KU198466	2
97L1793AFX-CAN-NT-AF-1997	1997	NA	Arctic fox	Canada	Arctic Bay	NT	NA	NA	no	KU198467	2
13N0473AFX-CAN-NU-AF-2013	2013	NA	Arctic fox	Canada	Resolute Bay	NU	NA	NA	no	KU198477	2
13N0643AFX-CAN-NU-AF-2013	2013	NA	Arctic fox	Canada	Grise Fiord	NU	NA	NA	no	KU198478	2
00N3340RFX-CAN-ON-RF-2000	2000	NA	Red fox	Canada	Walkerton	ON	NA	NA	ND	KU198468	2
01N10254RFX-CAN-ON-RF-2001	2001	NA	Red fox	Canada	Shelburne	ON	NA	NA	ND	KU198469	2
92N7894RFX-CAN-ON-RF-1992	1992	NA	Red fox	Canada	Swastika	ON	NA	NA	ND	KU198465	2
02N2980RFX-CAN-QC-RF-2002	2002	NA	Red fox	Canada	Salluit	QC	NA	NA	ND	KU198470	2
RV53-USA-F-1988	1988	NA	Fox	USA	NA	NA	NA	NA	ND	DQ010123	3
RV250-RUS-Squirrel-1983	1983	NA	*Citellus undulatus*	Russia	Tuvia	NA	NA	NA	ND	AY352480	3
RV1336-RUS-AF-1996	1996	NA	Arctic fox	Russia	Yakutia	NA	NA	NA	no	DQ010129	3

^1^ NA, not available

^2^ ND, not determined

### Sequencing protocol 1

#### RNA extraction

RNA was extracted from approximately 20 mg of brain tissue. To this end, the material was frozen in liquid nitrogen, homogenized using the Mikro-Dismembrator S (Sartorius, Göttingen, Germany), and the homogenate was suspended in 2 ml Buffer AL (Qiagen, Hilden, Germany) pre-heated to 56°C. The resulting suspension was mixed with 3 volumes of TRIzol LS Reagent (Life Technologies, Carlsbad, California, USA) and 0.2 volumes chloroform (Carl Roth GmbH + Co. KG, Karlsruhe, Germany) and after addition of 1 volume of 100% ethanol to the aqueous phase RNA was extracted using the RNeasy Mini Kit (Qiagen) as per the manufacturer’s instructions including an on-column DNase I digestion. If necessary, extracted RNA was concentrated with Agencourt RNA Clean XP magnetic beads (Beckman Coulter, Fullerton, USA). RNA quantity was determined using the Nanodrop ND1000 UV spectrophotometer (Peqlab, Erlangen, Germany).

#### cDNA-synthesis, library preparation and sequencing

cDNA was generated from total RNA with the cDNA synthesis system kit (Roche, Mannheim, Germany) and random hexamer primers (Roche) according to the Genome Sequencer RNA rapid library preparation manual (Roche). The resulting cDNA was fragmented to a target size of 300 bp using a Covaris M220 instrument (Covaris, Brighton, United Kingdom) and subsequently transformed to barcoded sequencing libraries using Illumina compatible adapters (Bioo Scientific Corp., Austin, USA) on a SPRI-TE library system (Beckman Coulter) with SPRIworks Fragment Library Cartridge II (Beckman Coulter) without size selection. After manual size selection with Agencourt AMPure XP magnetic beads (Beckman Coulter) for a target peak size of 350 bp, library quality was assessed using a Bioanalyzer 2100 instrument (Agilent Technologies, Böblingen, Germany) with a High Sensitivity DNA kit (Agilent Technologies). Finally, the libraries were quantified with the KAPA Library Quantification Kit for Illumina (Kapa Biosystems, Cape Town, South Africa) on a CFX96 Real-Time System (Bio-Rad Laboratories, Hercules, USA) and sequenced using the Illumina MiSeq instrument with MiSeq reagent kit v2 (Illumina, San Diego, USA) in 2 x 250 bp mode.

#### Sequence assembly

For sequence assembly, reads representing the respective sequences were selected by mapping the complete data set to reference sequences using the Genome Sequencer software suite (v2.6; Roche). Subsequently, the sorted reads were used for a de novo assembly using newbler (Roche) and the complete raw data sets were mapped along the resulting consensus sequences in order to identify potential sequencing errors. Thereafter, the sequences were visually inspected using Geneious (v6.1.7; Biomatters, Auckland, New Zealand).

### Sequencing protocol 2

#### RNA extraction

RNA was extracted from approximately 100 mg of brain tissue using TRIzol reagent as per the supplier’s instructions (Life Technologies).

#### cDNA-synthesis, library preparation and sequencing

A protocol for efficient RT-PCR amplification of the entire viral genome as a small number of overlapping amplicons was employed (Primers and protocols are available upon request). Amplicons derived from a single sample were pooled in equimolar concentrations and then used to generate a sequencing library using a Nextera XT kit as per the manufacturer’s directions (Illumina). Libraries were sequenced on an Illumina Miseq instrument using a MiSeq reagent kit v2 in 2 x 250 bp mode.

#### Sequence assembly

Sequence reads were assembled with the Lasergene software (v11; DNASTAR, Madison, Wisconsin USA). The paired end fastq files for each sample were assembled using a template-based method. Base coverage >200 was obtained throughout the genome with the exception of a small stretch of bases at the genomic termini for which coverage was very limited, regions which could not be unambiguously sequenced in any event due to their use as targets for amplification primers. As these regions tend to be highly conserved in all rabies viruses this was not considered to be a significant limitation. Complete assembled genomes were exported in fasta format for subsequent alignment and phylogenetic analysis as detailed below.

### Sequencing protocol 3

#### RNA extraction, cDNA-synthesis, library preparation and sequencing

RNA from brain material stored at -80°C was prepared for Next Generation Sequencing on the MiSeq platform (Illumina). Briefly, TRIzol (Life Technologies) extracted viral RNA was depleted of host genomic DNA and rRNA as described previously [22]. Double stranded (ds) cDNA was synthesized from 50 ng RNA, using a random cDNA synthesis system (Roche), according to the manufacturers’ instructions. The ds cDNA was purified using Ampure XP magnetic beads (Beckman Coulter) and 1ng used for the Nextera XT DNA sample preparation kit (Illumina). A sequencing library was prepared according to the manufacturers’ instructions and sequenced on an Illumina MiSeq with 2 x 150 bp paired end reads following standard Illumina protocols.

#### Sequence assembly

The total reads were mapped to an appropriate reference sequence in Burrows-Wheeler Alignment Tool (BWA, http://bio-bwa.sourceforge.net) using a script to generate intermediate consensus sequences in which any indels relative to the original reference sequence were appropriately called, then visualized in Tablet [23] as described previously [24].

### Sequence determination of arctic fox mitochondrial genes

For the determination of the genetic diversity of the reservoir host, mitochondrial (mtDNA) reference genes of arctic foxes, i.e. ATP6, ATP8, COX1, COX2, COX3, CYTB, ND1, ND2, ND3, ND4, ND4L and NDS [25] were selected from the International Nucleotide Sequence Database (INSDC) databases. Additionally, the mitochondrial D-Loop sequence as suggested before [26] was also used for mapping. Briefly, raw reads from sequencing were mapped along the reference genes and all reads identified as fox mitochondrial sequences were assembled de novo. Subsequently, the resulting consensus sequence was used as reference to map all reads of the dataset in order to identify potential sequencing errors. The resulting sequences were inspected visually in Geneious (v6.1.7; Biomatters).

### Evolutionary analyses

All obtained complete RABV genome sequences were aligned using ClustalW [27] (http://www.clustal.org) as implemented in Geneious (Biomatters), the sequences were trimmed to equal length and labelled with the collection year. Subsequently, phylogenetic analyses were performed using the Bayesian Markov Chain Monte Carlo (MCMC) simulation in the BEAST (Bayesian Evolutionary Analysis Sampling Trees) package v1.8.2 [28]. Selection of the evolutionary model using IQ-Tree (v1.1.0, [29] proposed use of the General Time Reversible model with rate heterogeneity (GTR + G). This model was used for MCMC simulation together with a relaxed molecular clock model and Bayesian Skyline population for 100,000,000 iterations, sampling every 10,000 states to give effective sample sizes. Maximum clade credibility trees (MCC) were annotated using TreeAnnotator (v1.8.2), 10% of the trees were removed as burn-in. The resulting final trees were visualized using FigTree (v1.4.2; http://tree.bio.ed.ac.uk/software/figtree/).

For phylogenetic analysis of the RABV Nucleoprotein (N) gene sequences, the dataset was extended with additional sequences from the INSDC databases (Table 2). All calculations were performed as described above for the full genome sequences except that for MCMC simulation the transitional model with rate heterogeneity (TIM + G) model was used.

**Table 2:** Details of additional sequences used for phylogenetic analyses of complete N gene.

Isolate name project	Location	Year	Host	INSDC accession no.	References
AY352458.1-RUS.Chabarovsk-RD-1980	Russia/Chabarovsk	1980	Raccon dog	AY352458.1	[60]
AY352459.1-RUS.Chita-SF-1977	Southern Siberia	1977	Corsac fox	AY352459.1	[60]
AY352462.1-RUS.Norilsk-H-1998	Russia/Norilsk	1998	Human (ex wolf)	AY352462.1	[60]
AY352474.1-RUS.Pskov-RF-1990	Russia/Pskov	1990	Red fox	AY352474.1	[60]
AY352487.1-RUS-SA-1986	Russia/Yakutia	1986	Arctic fox	AY352487.1	[60]
AY352491.1-KAZ-RF-1988	Kazakhstan	1988	Red fox	AY352491.1	[60]
AY352498.1-USA-AK-D-1988	Alaska	1988	Dog	AY352498.1	[60]
AY352500.1-USA-AK-RF-1988	Alaska	1988	Red fox	AY352500.1	[60]
AY730596.1-KOR-D-2004	South Korea	2004	Dog	AY730596.1	[61]
AY956319.1-IND-H-2004	India	2004	Human	AY956319.1	-
EF611828.1-RUS-SA-1987	Russia/Yakutia	1987	Arctic fox	EF611828.1	[17]
EF611830.1-RUS-SA-1987	Russia/Yakutia	1987	Arctic fox	EF611830.1	[17]
EF611843.1-USA-AK-RF-2007	Alaska	2007	Red fox	EF611843.1	[17]
EF611848.1-USA-AK-RF-2008	Alaska	2008	Red fox	EF611848.1	[17]
EF611851.1-USA-AK-AF-2006	Alaska	2006	Arctic fox	EF611851.1	[17]
EF611853.1-USA-AK-D-2006	Alaska	2006	Dog	EF611853.1	[17]
EF611855.1-USA-AK-D-2006	Alaska	2006	Dog	EF611855.1	[17]
EF611868.1-SouthernSiberia-D-1980	Southern Siberia	1980	Dog	EF611868.1	[17]
JX944565.1-NPL-H-2003	Nepal	2003	Human	JX944565.1	[20]
JX944601.1-NPL-M-2010	Nepal	2010	Mongoose	JX944601.1	[20]
JX987745.1-GRL-D-1980	Greenland	1980	Dog	JX987745.1	[20]
L20675.2-CAN-ON-AF-1991	Canada/Ontario	1991	Arctic fox	L20675.2	[62]
U03769.1-CAN.Arctic-D-1993	Canada/Arctic	1993	Dog	U03769.1	[63]
U03770.1-CAN.HudsonBay-D-1992	Canada/Hudson Bay	1992	Dog	U03770.1	[63]
U11735.1-CAN-ON-RF-1993	Canada/Ontario	1993	Red fox	U11735.1	[63]
U22474.1-France-RF-1991	France	1991	Red fox	U22474.1	[15]
U22477.1-MEX-D-1991	Mexico	1991	Dog	U22477.1	[15]
U22654.1-GRL-AF-1981	Greenland	1981	Arctic fox	U22654.1	[15]
U42705.1-F.R.Yugoslavia-cattle-1981	F.R.Yugoslavia	1981	Cattle	U42705.1	-
U42432.1-EST-RD-1991	Estonia	1991	Raccoon dog	U43432.1	-

From 55 of 58 Greenland samples, the substitutions per site and year were determined for the whole genome and all five protein coding nucleotide-sequences (N-, P-, M-, G- and L- Gene), respectively. Sequences were aligned and best fitting evolutionary models selected as described above. The best model for the N and P genes was Kimura 3-parameter (K81, [30], for the G and L genes Kimura 3-parameter with unequal frequencies (K81uf), for the M gene Kimura 2-parameter (K2P, [31], and for the whole genome Kimura 3-parameter with unequal frequencies and proportion of invariable sites (K81uf + I). Beast analysis was performed as described above except for the N gene for which 2,000,000,000 iterations and samples every 200,000 states were needed.

To infer the evolutionary and phylogenetic relationships of foxes, the sequences were aligned using ClustalW and subsequently phylogenetic trees were calculated using the maximum likelihood (ML) method as implemented in MEGA (v5.2; [32]). For these calculations, the best fitting evolutionary model, Tamura and Nei 1993 with rate heterogeneity (TN93 + G), was selected using MEGA’s model test function and 1000 bootstrap replicates were calculated. The resulting phylogenetic trees were visualized in MEGA.

### Geographical mapping

**Fig 1:**
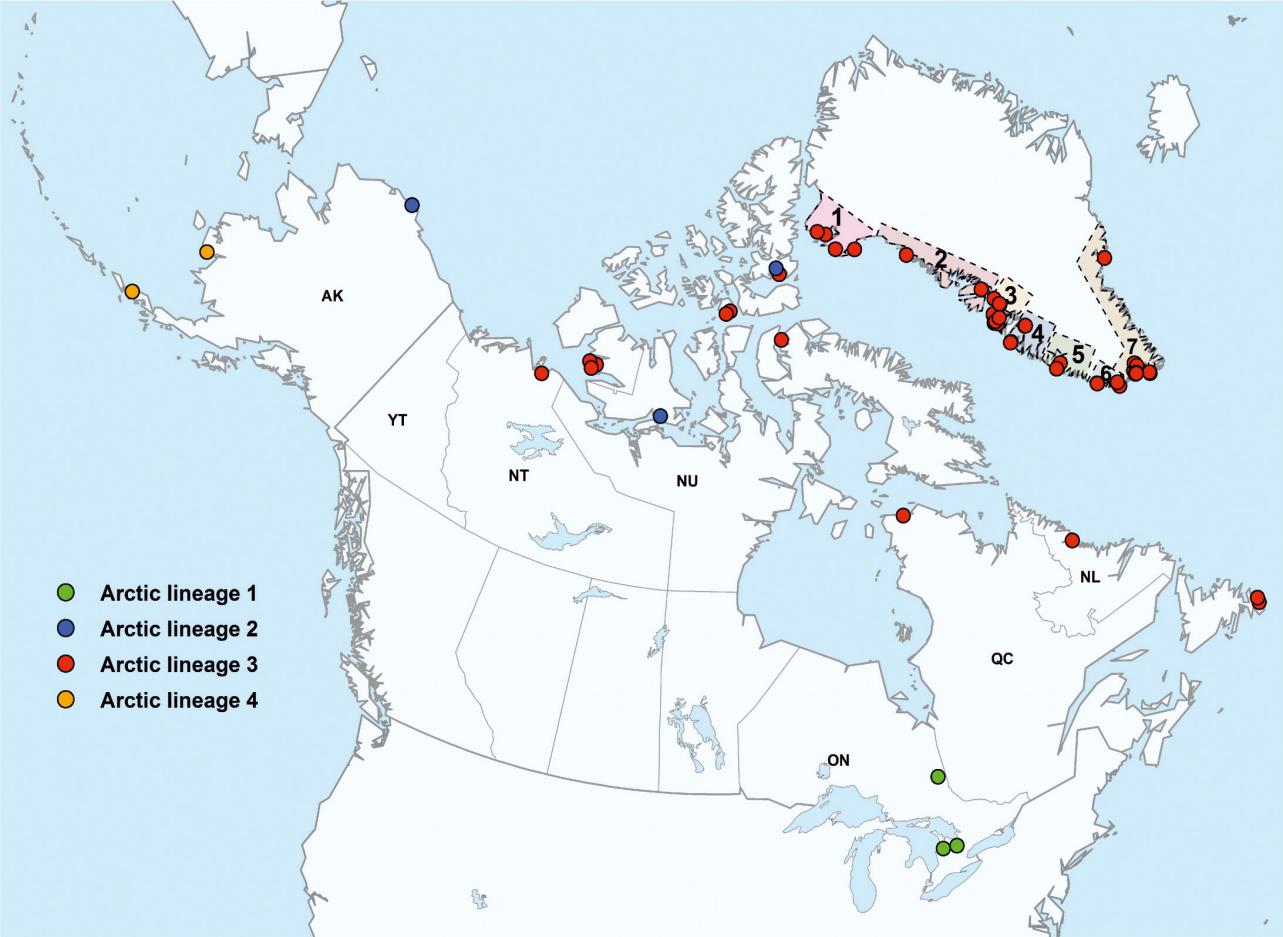
Map illustrating the origin of samples from Greenland, Canada and Alaska and their assignment to arctic lineages (Green = arctic lineage 1; Blue = 2; Red = 3; Yellow = 4). Numbers in Greenland represent regions, while for Canada and Alaska postal abbreviation of the province/territory/states is indicated.

Approximate sampling locations in Greenland, Canada and the USA (Table 1, Fig 1) were visualized using ArcGis 10.0 (ESRI) at the highest spatial resolution available.

## Results

To enable in-depth phylogenetic analysis, viral sequences encoding all genes were generated for all arctic RABV samples (S1 Table, Fig 1). For Greenland fox samples, complete coding sequences of 12 mitochondrial genes were determined from the available host sequence data in order to relate the viral phylogeny to fox populations and to detect potentially existing spatial fox distribution patterns.

### Greenland RABV segregation into distinct arctic-3 subclades

**Fig 2:**
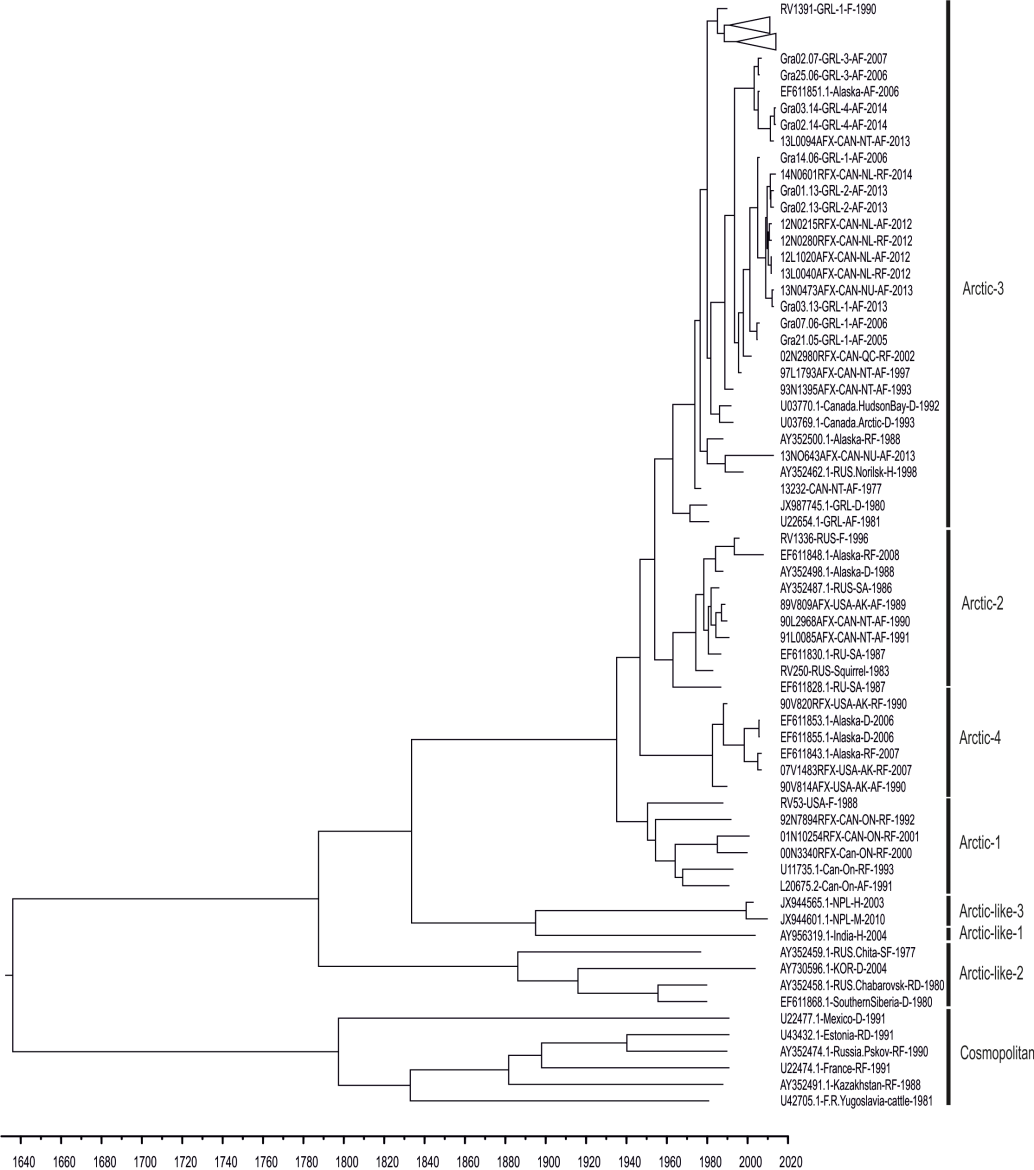
Time resolved phylogenetic tree of the RABV complete N gene (maximum clade credibility (MCC) calculated with BEAST from a total of 109 RABV sequences (Tables 1 and 2). RABV genetic lineages are labelled to pre-defined clusters according to a previous study [17].

To classify the sequenced viruses globally into pre-defined lineages, only complete N gene sequences (n = 109, Tables 1 and 2) were used, since to date no full-genome sequences are available for the Arctic rabies strains. All sequenced Greenland RABV (Table 1) clustered within the previously established circumpolar arctic-3 lineage (Fig 2). This lineage appears to be rather heterogeneous. Older arctic-3 viruses from Greenland (obtained in 1980/81) are clearly separated from recent Greenland RABVs.

**Fig 3:**
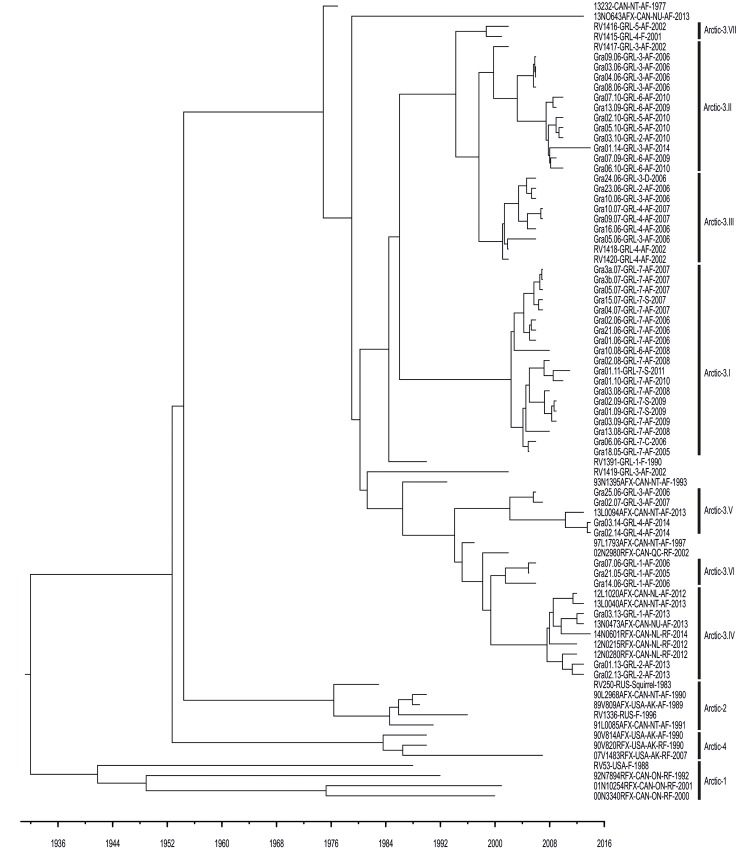
Maximum clade credibility (MCC) phylogenetic tree using complete genome sequences from 79 RABV samples sequenced in this study (Table 1). The minimum identity of sequences to be grouped into a single subclade within arctic lineage 3 was set to 99.5%.

For higher resolution of the heterogeneous arctic-3 lineage the analysis was repeated using full-genome sequences obtained for 79 RABV samples from Greenland, Canada, USA (Alaska) and Russia between 1977 and 2014 (Table 1). This high resolution analysis confirmed previous delineation of the arctic RABV strain into four major lineages (arctic 1–4, Figs 1 and 3), whereby RABV from Greenland exclusively grouped into arctic-3. Furthermore, it enabled the distinction of seven subclades and seven outliers (1977–2013) within arctic-3 (Fig 3), with the most recent common ancestor (MRCA) of the analyzed viruses (Fig 3) occurring approximately 82 years ago (95% HPD values, 73–92 years). The MRCA for the Greenland samples within this dataset dates back circa 35 years (95% HPD values, 32–37), whilst the divergence into subclades occurred between 15 years (subclade 3.VII; 95% HPD values, 14–17 years) and 6 years (subclade 3.IV; 95% HPD values, 5–8 years) ago.

Of these seven subclades, only arctic-3.IV and 3.V contained viruses from both Greenland and Canada (2006 to 2014). Greenland RABV from arctic-3.IV -3.VI (n = 10) originated from north-western and western Greenland (regions 1–4, Fig 1), collected between 2005 and 2014. Greenland specific subclades 3.II, 3.III, and 3.VII consisted of 24 RABV from the western coastline (regions 2–6) obtained between 2001 and 2014. In contrast, all but one RABV within subclade 3.I (2005–2011) originated from southern Greenland (region 7), with Gra10.08-GRL-6-AF-2008 being the only exception collected in region 6 (Figs 1 and 3).

### Partial sequencing overestimates substitution rates

Both the individual gene sequences and the full-genome alignment were used to estimate the mean nucleotide substitution rate utilizing BEAST [28]. For the N gene sequences (Tables 1 and 2, Fig 2) an estimate of 2.5 E-4 substitutions per site per year (95% high posterior density (HPD), 1.9 E-4–3.1 E-4) was obtained. When only N gene sequences from Greenland (N = 55) were considered, the estimate was 3.1 E-4 substitutions per site per year, a value in the range of previous studies. Among the different genes, lowest and highest substitution rates were observed in the L gene and the P gene, respectively (Table 3). The substitution rate of 2.5 E-4 per site per year (95% HPD values, 2.1 E-4–3.0 E-4) for the full genome sequence is similar to the value observed for the L gene.

**Table 3:** Comparison of substitution rates per site of RABVs.

References	Number of sequences	Whole genome	Substitution per site (95% HPD)					Comment
			N -gene	P—gene	M- gene	G—gene	L—gene	
This study	55	2.5	3.1 E-4	6.0 E-4	4.4 E-4	3.3 E-4	2.4 E-4	Whole genome sequences of this study (Greenland only)
		(2.1 E-4–3.0 E-4)	(1.8 E-4–4.4 E-4)	(4.2 E-4–7.9 E-4)	(2.7 E-4–6.4 E-4)	(2.2–4.5 E-4)	(1.9–2.9 E-4)	
	79	2.2 E-4						Whole genome sequences of this study
		(2.0 E-4–2.5 E-4)						
	109		2.5 E-4					N gene sequences (Fig 2)
			(1.9 E-4–3.1 E-4)					
[17]	32		1.23 E-4					Arctic/Arctic-like RABV
			(6.8 E-5–1.83 E-4)					
	17		3.89 E-4					European red fox
			(5.1 E-5–6.7 E-4)					
	60		1.48 E-4					Combination of arctic/arctic-like, European red fox and 11 further isolates
			(8.1 E-5–2.23 E-4)					
[64]	76				4.6 E-4			Chinese street RABV
					(2.5 E-4–6.6 E-4)			
[65]	N = 151		2.3 E-4			3.9 E-4		Global genetic diversity of RABV for which the time(year) of sampling was available
	G = 74		(1.1 E-4–3.6 E-4)			(1.2 E-4–6.5 E-4)		
[20]	67		3.8 E-4					Arctic—related RABV
			(2.3 E-4–5.4 E-4)					
[66]	N = 80		5.27 E-4			4.1 E-4		N = 1350 bp
	G = 71		(± 0.23 E-4)			(± 0.3 E-4)		G = 690 bp
[18]	212		3.817 E-4					Arctic RABV
			(2.816 E-4–4.825 E-4)					N = 500 bp

### Greenland arctic foxes segregate into three major genetic clusters

**Fig 4:**
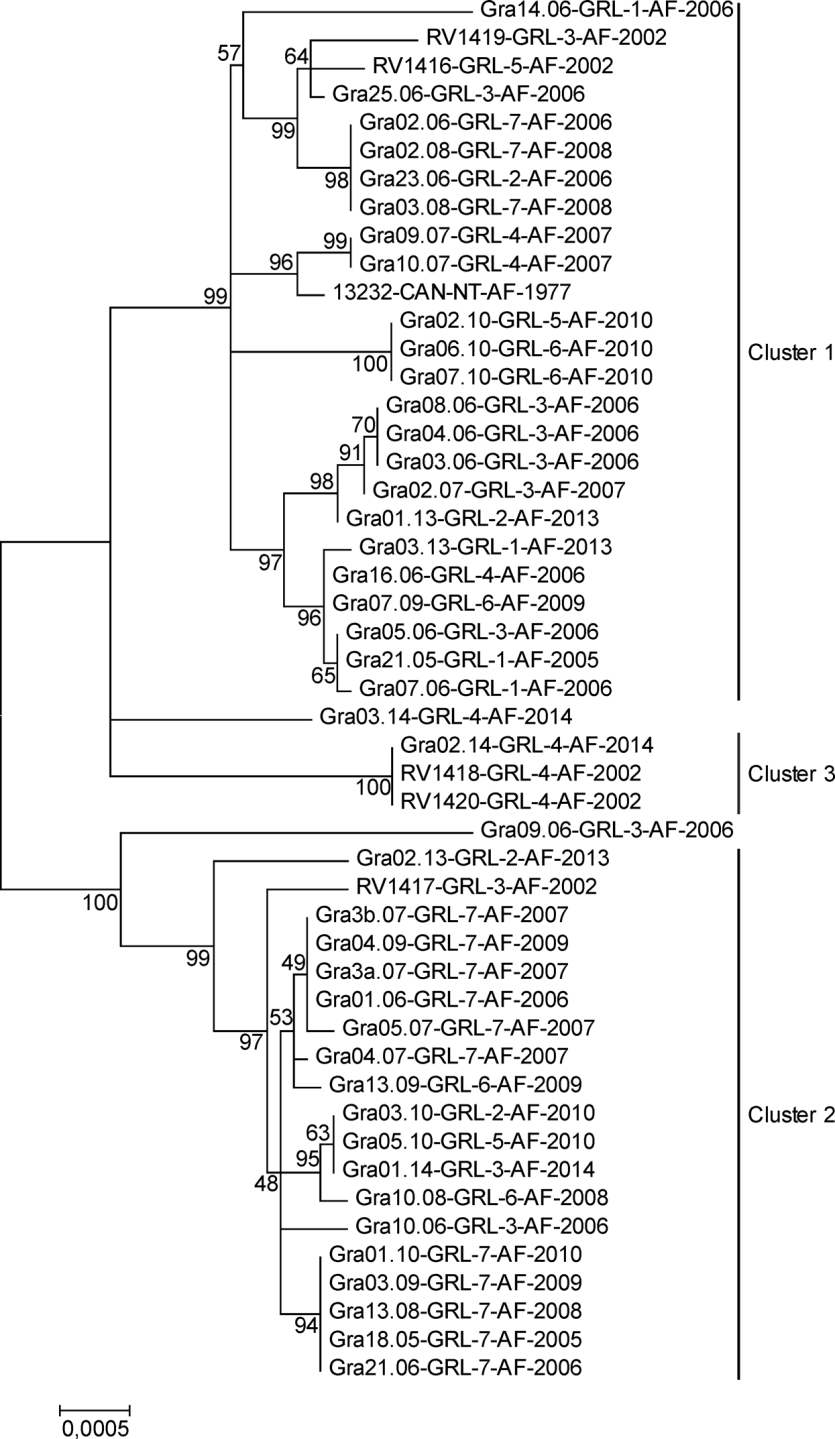
Phylogram of 49 arctic foxes generated from concatenated sequences of 12 mitochondrial protein coding sequences (ATP6, ATP8, COX1, COX2, COX3, CYTB, ND1, ND2, ND3, ND4, ND4L and NDS). Bootstrap values higher than 40% are indicated. The minimum identity of sequences to be grouped into a fox cluster was set to 99.7%.

Direct untargeted NGS determined by protocols 1 and 3 from original rabies positive brain samples from Greenland (n = 48) and Canada (n = 1) yielded a substantial amount of host sequences. This provided the unique opportunity to analyze the genetic structure of the arctic fox population. Analysis of 12 mitochondrial gene sequences revealed three major maternal lineages and additional outliers (Fig 4). Genetic identity within the main clusters was between 99.7% and 100% (Cluster 1), 99.8% and 100% (Cluster 2), and 99.9% (Cluster 3); while identity between clusters was between 99.4% and 99.6%. For all clusters there was a distinct, partly overlapping (cluster 1 and 2) geographical distribution. Twenty-one animals of cluster 1 were detected in North-western and Western Greenland (regions 1–6), while three foxes originated from the far distant Southern coast (region 7). Interestingly, a fox sample from the Northwestern Territories of Canada obtained in 1977 was almost identical with two foxes collected in 2007 in Western Greenland (Fig 1, Table 1). Half of the arctic foxes from cluster 2 (n = 19) originated from southern Greenland (region 7), while the remaining foxes were located in the western parts of the island (regions 2, 3, 5, and 6). The highly conserved cluster 3 comprised exclusively of arctic foxes from region 4, with samples from 12 years apart (Fig 4). The outliers originated from the western regions 2, 3, and 4 (Figs 1 and 4).

### Occurrence of RABV variants in different fox clusters

**Fig 5:**
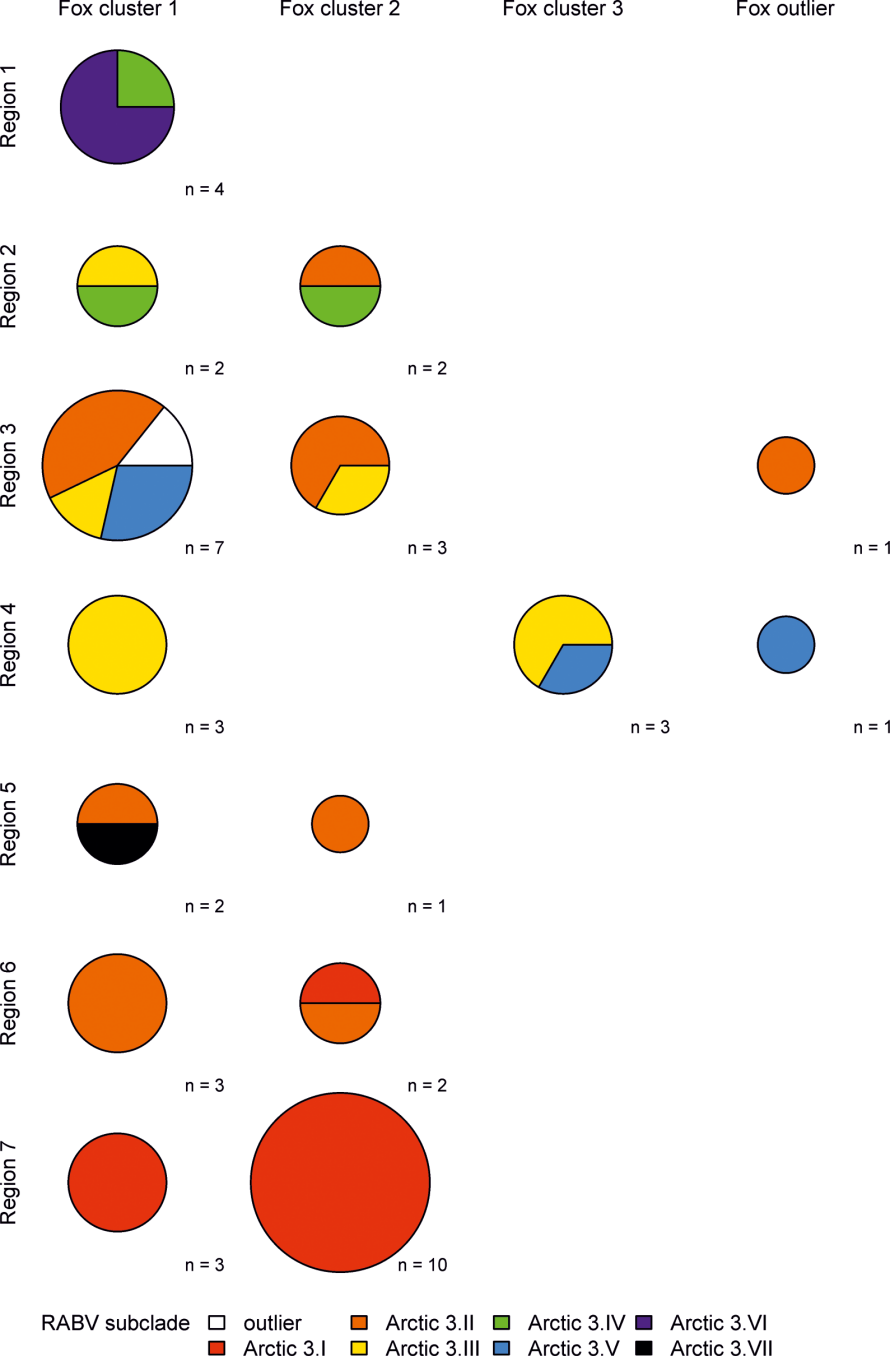
Comparison of landscape genetics of Greenland arctic foxes (Figs 1 and 4) and their RABV subclades (Fig 3). The size of circles represents the number of observations.

Combinatorial analysis of RABV subclades, fox mitochondrial genes and the geographic origin revealed no association (Fig 5). The majority of samples were assigned to fox cluster 1 which was detected in all regions and comprised all RABV subclades. Fox cluster 2 was restricted mainly to the southern regions and therefore mostly infected with RABV subclades arctic 3.I and 3.II. Fox cluster 3 in contrast is only found in region 4, but represents 2 different RABV subclades (Figs 3 and 5).

## Discussion

For a long time, there has been limited knowledge on the epidemiology of arctic rabies in Greenland [9–12]. It was only recently that the temporal occurrence, spatial distribution, and spread of arctic rabies in Greenland was investigated based on historical observations [13], however, this study did not provide any context to the RABV circulating on the island. While previous molecular epidemiological studies on RABV from Greenland using partial N gene sequences revealed that they belong to the arctic-3 lineage [16,17], unfortunately, the number of samples and the sequence length examined prevented further in-depth phylogenetic analyses. However, with the advent of NGS the determination of whole genome sequences for molecular epidemiological studies of RABV has become more efficient and comprehensive as illustrated recently for skunk variant of RABV from California [33].

Here, NGS derived sequence analysis is simultaneously applied to both arctic RABV strains and arctic fox genetics. A large number of RABV full genome sequences and mitochondrial genes were utilized for the phylogenetic analysis of arctic RABVs and their respective arctic fox host species.

This dataset comprises a comprehensive number of RABV samples of the arctic strain (n = 79), particularly from Greenland (n = 55), thereby providing an improved knowledge of the molecular evolution of the circulating viruses and a more comprehensive understanding of the historical perspective of the disease across the Arctic. One limitation of this and previous studies is that collection of samples within Greenland, an island comprising 2.1 million km², relies on a passive surveillance system that results in uneven distribution of submissions, i.e. samples are only submitted from areas where close fox-human encounters are possible and there is a lack of samples from central and eastern parts of Greenland (Fig 1). For instance, in sparsely populated east Greenland only 12 cases have been observed since the late 1960s until 2014 [13], suggesting either low level of infection or underreporting [17,34]. Whilst in previous studies only selected RABV were considered [16–18], here nearly all viruses from rabies cases detected between 2005 and 2014 were included, together with selected samples from an earlier study [16] that were available for re-analysis using NGS.

While in Alaska lineage 4, in Southern Canada lineage 1 and in Russia mainly RABV of arctic lineage 2 circulate, interestingly, both historic and recent RABV from Greenland exclusively fall within arctic-3 lineage (Fig 1). At present, based on our sample set of complete genome sequences, at least seven distinct subclades (arctic 3.I–3.VII) within this lineage have been circulating in Greenland (Fig 3). This delineation, however, contrasts results of a previous study, in which many of the Greenland samples (1990–2002) only clustered as one separate sub-group within arctic lineage 3 [18]. This can be explained by the limited length of the partial N gene sequence (500 bp) used which prevents a higher resolution of the tree. In fact, combining all previous partial N gene sequences (S2 Table) [16–18] with our dataset resulted in a rather limited alignment of 163 nucleotides in length which is too short to support phylogenetic analysis (S1 Fig). These limitations confound attempts to compare the studies and put them into a larger perspective, both temporally and spatially. Therefore, in this study this issue was addressed as best as possible by obtaining full length genome sequences from a subset of a previous study [16].

From a geographical perspective, the arctic-3 subclades defined here showed certain localized distributions (Fig 5). Considering the large home ranges of arctic foxes in connection with long distance migration of more than 1000 km [35–37], a larger geographical overlap would have been expected. However, this holds true only for subclade 3.II, the oldest cluster with viruses collected between 2002 and 2014. Phylogenetic analyses showed that all RABV from Greenland analyzed in this study have derived from Canadian incursions (Fig 3) with the oldest sample from 1977 having the most basal position in the tree. In contrast, an incursion and further spread of arctic RABV from Svalbard across the Greenland Sea into eastern and northeastern island as suggested previously [11] is not evident in this present study. In fact, Svalbard RABVs were shown to be more closely related to arctic RABVs from Russia [17,19].

The fact that subclades arctic-3.IV and 3.V are the only subclades to be identified on the North American mainland while all other subclades are restricted to Greenland’s western and southern parts, respectively, may represent evidence for a more recent exchange of viruses (Figs 1 and 3). In fact, these subclades 3.IV and 3.V were found in regions of Greenland where the distance to neighboring Ellesmere Island, Canada, is shortest. Here, pack ice that frequently bridges these two land masses may facilitate the spread of the disease [16,18]. Hence, the Smith Sound, the uninhabited sea passage between Greenland and Canada’s northernmost islands may play an important role in the exchange of arctic RABVs in this part of the Arctic [38]. This is supported by the close genetic relation between RABV Gra03.13-GRL-1-AF-2013 and 13N0473AFX-CAN-NU-AF-2013, with nearly identical genomes (single substitution), despite the fact that they are geographically separated by a distance of 750 km (Thule Air Base to Resolute Bay).

In Greenland, viruses with a high nucleotide substitution rate may have evolved into younger subclades that have a narrower geographical distribution and are only identified on the island (Figs 3 and 5). For instance, arctic subclade 3.I is only found in the southern parts (Region 6,7; Fig 5). Interestingly, the most ancestral RABV still circulating (subclade arctic 3.II) has the widest geographic spread across the entire western part of Greenland. Such spread has also been demonstrated epidemiologically [13]. The dynamic observed here is further demonstrated by the observation that older subclades previously found in Greenland (Fig 2), [15–18] seem to have disappeared.

It is interesting that arctic lineage 2 (Figs 1 and 3) had not (yet) been detected in Greenland, particularly considering that other arctic lineages appear to have spread [18]. As a case in point, one sample from 1990 from Grise Fjord, which has close proximity to Greenland, was arctic lineage 2, while a sample from the same place twenty-three years later was arctic 3 (Figs 1 and 3, Table 1). As regards the other arctic lineages, lineage 4 seems to be restricted to Alaska [18,39] (Fig 1), while circulation of arctic lineage 1 in Southern Ontario, Canada is highly restricted due to oral rabies vaccination [40,41].

The observed genetic dynamic within arctic RABVs is also demonstrated by the nucleotide substitution rates inferred from this dataset at the full genome level. Although in comparison with partial sequence analysis of other RABVs this appears low (Table 3), a substitution rate of 2.5 E-4 per site and year still indicates a substantial evolutionary dynamic. Discrepancies in the substitution rates and in the resulting MRCA as discussed before [18], are a result of the respective partial sequence used for calculation (Table 3). Thus, not all RABV genes are equal for evolutionary analyses, as previously suggested [42,43]. Taken together, use of complete genome sequences should result in a more accurate substitution rate, closely reflecting the actual virus evolution and genetic dynamic.

The use of unbiased NGS offered the unique opportunity to simultaneously obtain reservoir host derived RNA sequences from the same sample for population analysis. Similar to previous studies [26,44] we initially used D-Loop sequences for this analysis. However, this did not allow a clear distinction because of the high genetic identities observed. Instead, for increased resolution, the 12 protein-coding mitochondrial genes enabled delineation of Greenland’s fox population into three main genetic clusters as shown recently [39]. Still, the high genetic identity both within and between the three main genetic fox clusters ranging between 99.4% and 100% may support assumptions that the genetic diversity of island arctic foxes compared to main land populations as a result of colonization is low [37,45]. The detection of three almost genetically identical arctic fox samples from the Northwestern Territories of Canada and Western Greenland (Fig 1) three decades apart may be evidence for high gene flow among arctic fox subpopulations, contributing to low genetic differentiation at least in the mitochondrial genome and further corroborate this hypothesis. In contrast, the results from this present study of mitochondrial gene analysis are in agreement with previous observations showing a fine-scale spatial population structure in Alaskan arctic foxes [39]. Future more detailed comparative haplotype analysis of arctic foxes including nuclear loci as well as fox genetic data from Canada’s northern territories and Alaska should corroborate this fact.

While previously, mtDNA structure in arctic and red foxes from Alaska did not correspond to RABV variant structure in either species, microsatellite analyses identified 3 and 4 groups of arctic foxes, closely matching the distribution of rabies virus variants in the state [39]. Although the Greenland RABV arctic-3 subclades were not evenly distributed among the different mitochondrial fox clusters (Fig 5), these data provide no evidence for independent isolated evolutionary development of RABV in different arctic fox lineages but rather resemble geographic separation.

In our study we focused on mitochondrial DNA (mtDNA) alone for characterization of the population genetics of arctic foxes. Analysis of mtDNA reveals a longer-term view of population structure than do fast evolving nuclear markers (e.g., microsatellites) and analysis of multiple mitochondrial genes, as in this study, yields finer resolution of relationship than studies focusing on a single gene. Nonetheless, any combination of mitochondrial genes is effectively considered a single locus. Recent studies included multiple markers of nuclear genes i.e. microsatellites to investigate host genetics in carnivores, sometimes resulting in conflicting results when compared to parallel investigated mtDNA [46–50]. However, a meta-analysis showed that mtDNA was robust in determining patterns of population history and yielded similar results to microsatellites [51]. Finally, the lack of a standardized approach for using both types of marker genes and the limited information on nuclear genes to those that are expressed as mRNA and are thus part of the dataset precluded further analyses. For instance, in a recent study the transcriptome was used for evolutionary studies of Arctic and red foxes [52]. While this information should be available it is unclear whether it would be sufficient for intra-species genetic studies.

## Conclusion

By combining full length RABV genome sequence analysis and host derived sequences the interaction of viruses and their hosts was exemplarily demonstrated and may serve as a model approach for analysis of real-world understanding of infectious disease dynamics and virus-host interdependencies using a landscape genetics approach as suggested for dog mediated rabies [53]. In this study, although no interdependencies based on mtDNA were identified, nevertheless this approach led to a better understanding of the evolution, dynamics and geographical spread of arctic rabies in Greenland.

A high degree of genetic identity both of RABVs and arctic foxes from Canada and Greenland suggests the movement of infected animals between the two landmasses. The overall diversity of arctic RABV in Greenland was very limited and only by analyzing the entire genome, a high resolution of the genetic evolution was possible, providing real-time insights into viral evolution. These results may be useful for future control strategies of arctic fox rabies. In contrast to previous statements [13], given the unique geographical location of Greenland, the expected reduction of connectivity by pack-ice due to climate change [54,55] and the geographic separation of individual host and virus genetic subclades despite long distance movement [36], the idea of arctic rabies control using oral rabies vaccination (ORV) in selected coastal areas appears feasible [56,57]. While preliminary field trials in Newfoundland (Canada) [58] and even northern Greenland [59] demonstrated in principle that ORV could be undertaken in remote northern regions, a targeted vaccination strategy would have to be developed before an elimination program could be implemented.

## Supporting Information

S1 Table Overview of raw sequence data using NGS generated in this study. (PDF)

S2 Table Reference sequences used in S1 Fig. (PDF)

S1 Fig Phylogram combining all previous partial N gene sequences (S2 Table) [16–18] with our dataset based on a rather limited alignment of 163 nucleotides in length. (TIFF)

## References

[1] Dietzgen RG, Calisher CH, Kurath G, Kuzmin IV, Rodriguez LL, et al Family Rhabdoviridae In: King AM, Adams MJ, Carstens EB, Lefkowitz EJ, editors. Virus taxonomy: classification and nomenclature of viruses—Ninth Report of the International Committee on Taxonomy of Viruses. San Diego: Elsevier; 2012 pp. 686–713.

[2] World Health Organisation. Expert Consultation on Rabies, Second Report. World Health Organ Tech Rep Ser. 2013; 982: 1–150. 24069724

[3] World Health Organization. Report of A WHO/NVI Workshop on arctic rabies. Upsala, Sweden, 24–27 April 1990. 1–26 p.

[4] Noren K, Carmichael L, Dalen L, Hersteinsson P, Samelius G, et al Arctic fox Vulpes lagopus population structure: circumpolar patterns and processes. Oikos. 2011; 120: 873–885.

[5] Macdonald DW. The Ecology of Carnivore Social-Behavior. Nature. 1983; 301: 379–384.

[6] Eberhardt LE, Hanson WC, Bengtson JL, Garrott RA, Hanson EE. Arctic Fox Home Range Characteristics in an Oil-Development Area. J Wildl Manage. 1982; 46: 183–190.

[7] Anthony RM. Home ranges and movements of arctic fox (Alopex lagopus) in Western Alaska. Arctic. 1997; 50: 147–157.

[8] Frafjord K, Prestrud P. Home Range and Movements of Arctic Foxes Alopex-Lagopus in Svalbard. Polar Biol. 1992; 12: 519–526.

[9] Crandell RA. Arctic Fox Rabies In: Baer GM, editor. The Natural History of Rabies. New York: Academic Pr; 1975 pp. 23–40.

[10] Jenkins M, Wamberg K. Rabies discovered in Greenland. J Am Vet Med Assoc. 1960; 137: 183–185. 14407072

[11] Leisner K. Rabies in Greenland, 1975–2001. Rabies Bulletin Europe. 2002; 26: 10–14.

[12] Mork T, Prestrud P. Arctic Rabies—A Review. Acta Vet Scand. 2004; 45: 1–9. 15535081 10.1186/1751-0147-45-1 PMC1820997

[13] Raundrup K, Moshoj CM, Wennerberg SE, Kapel CMO. Spatiotemporal distribution of rabies in Arctic foxes in Greenland. Eur J Wildl Res. 2015; 61: 457–465.

[14] Crandell RA. Laboratory Investigation of Arctic Strains of Rabies Virus. Acta Pathol Microbiol Scand. 1965; 63: 587–596. 14324666 10.1111/apm.1965.63.4.587

[15] Kissi B, Tordo N, Bourhy H. Genetic Polymorphism in the Rabies Virus Nucleoprotein Gene. Virology 1995; 209: 526–537. 7778285 10.1006/viro.1995.1285

[16] Mansfield KL, Racloz V, McElhinney LM, Marston DA, Johnson N, et al Molecular epidemiological study of Arctic rabies virus isolates from Greenland and comparison with isolates from throughout the Arctic and Baltic regions. Virus Res. 2006; 116: 1–10. 16198016 10.1016/j.virusres.2005.08.007

[17] Kuzmin IV, Hughes GJ, Botvinkin AD, Gribencha SG, Rupprecht CE. Arctic and Arctic-like rabies viruses: distribution, phylogeny and evolutionary history. Epidemiol Infect. 2008;136: 509–519. 17599781 10.1017/S095026880700903X PMC2870842

[18] Nadin-Davis SA, Sheen M, Wandeler AI. Recent emergence of the Arctic rabies virus lineage. Virus Res. 2012; 163: 352–362. 10.1016/j.virusres.2011.10.026 22100340

[19] Johnson N, Dicker A, Mork T, Marston DA, Fooks AR, et al Phylogenetic comparison of rabies viruses from disease outbreaks on the Svalbard Islands. Vector Borne Zoonotic Dis. 2007; 7: 457–460. 17767407 10.1089/vbz.2006.0555

[20] Pant GR, Lavenir R, Wong FY, Certoma A, Larrous F, et al Recent emergence and spread of an Arctic-related phylogenetic lineage of rabies virus in Nepal. PLoS Negl Trop Dis. 2013; 7: e2560 10.1371/journal.pntd.0002560 24278494 PMC3836727

[21] Dean DJ, Abelseth MK, Athanasiu P. The fluorescence antibody test In: Meslin FX, Kaplan MM, Koprowski H, editors. Laboratory techniques in rabies. 4^th^ ed. Geneva: World Health Organization; 1996 pp. 88–93.

[22] Marston DA, McElhinney LM, Ellis RJ, Horton DL, Wise EL, et al Next generation sequencing of viral RNA genomes. BMC Genomics. 2013; 14: 444 10.1186/1471-2164-14-444 23822119 PMC3708773

[23] Milne I, Stephen G, Bayer M, Cock PJ, Pritchard L, et al Using Tablet for visual exploration of second-generation sequencing data. Brief Bioinform. 2013; 14: 193–202. 10.1093/bib/bbs012 22445902

[24] Marston DA, Wise EL, Ellis RJ, McElhinney LM, Banyard AC, et al Complete genomic sequence of rabies virus from an ethiopian wolf. Genome announc. 2015; 3: e00157–15. 10.1128/genomeA.00157-15 25814597 PMC4384137

[25] Delisle I, Strobeck C. A phylogeny of the Caniformia (order Carnivora)based on 12 complete protein-coding mitochondrial genes. Mol Phylogenet Evol. 2005; 37: 192–201. 15964215 10.1016/j.ympev.2005.04.025

[26] Dalen L, Fuglei E, Hersteinsson P, Kapel CMO, Roth JD, et al Population history and genetic structure of a circumpolar species: the arctic fox. Biol J Linn Soc. 2005; 84: 79–89.

[27] Thompson JD, Higgins DG, Gibson TJ. CLUSTAL W: improving the sensitivity of progressive multiple sequence alignment through sequence weighting, position-specific gap penalties and weight matrix choice. Nucleic Acids Res. 1994; 22: 4673–4680. 7984417 10.1093/nar/22.22.4673 PMC308517

[28] Drummond AJ, Suchard MA, Xie D, Rambaut A. Bayesian phylogenetics with BEAUti and the BEAST 1.7. Mol Biol Evol. 2012; 29: 1969–1973. 10.1093/molbev/mss075 22367748 PMC3408070

[29] Nguyen LT, Schmidt HA, von Haeseler A, Minh BQ. IQ-TREE: A Fast and Effective Stochastic Algorithm for Estimating Maximum-Likelihood Phylogenies. Mol Biol Evol. 2015; 32: 268–274. 10.1093/molbev/msu300 25371430 PMC4271533

[30] Kimura M. Estimation of evolutionary distances between homologous nucleotide sequences. Proc Natl Acad Sci U S A. 1981; 78: 454–458. 6165991 10.1073/pnas.78.1.454 PMC319072

[31] Kimura M. A simple method for estimating evolutionary rates of base substitutions through comparative studies of nucleotide sequences. J Mol Evol. 1980; 16: 111–120. 7463489 10.1007/BF01731581

[32] Tamura K, Peterson D, Peterson N, Stecher G, Nei M, et al MEGA5: Molecular Evolutionary Genetics Analysis Using Maximum Likelihood, Evolutionary Distance, and Maximum Parsimony Methods. Mol Biol Evol. 2011; 28: 2731–2739. 10.1093/molbev/msr121 21546353 PMC3203626

[33] Borucki MK, Chen-Harris H, Lao V, Vanier G, Wadford DA, et al Ultra-deep sequencing of intra-host rabies virus populations during cross-species transmission. PLoS Negl Trop Dis. 2013; 7: e2555 10.1371/journal.pntd.0002555 24278493 PMC3836733

[34] Prestrud P, Krogsrud J, Gjertz I. The Occurrence of Rabies in the Svalbard Islands of Norway. J Wildl Dis. 1992; 28: 57–63. 1548803 10.7589/0090-3558-28.1.57

[35] Goltsman M, Kruchenkova EP, Sergeev S, Johnson PJ, Macdonald DW. Effects of food availability on dispersal and cub sex ratio in the Mednyi Arctic fox. Behav Ecol Sociobiol. 2005; 59: 198–206.

[36] Tarroux A, Berteaux D, Bety J. Northern nomads: ability for extensive movements in adult arctic foxes. Polar Biol. 2010; 33: 1021–1026.

[37] Noren K, Carmichael L, Fuglei E, Eide NE, Hersteinsson P, et al Pulses of movement across the sea ice: population connectivity and temporal genetic structure in the arctic fox. Oecologia. 2011; 166: 973–984. 10.1007/s00442-011-1939-7 21344255

[38] Charles E. Movements of Arctic Fox populations in the region of Baffin Bay and Smith Sound. Polar Rec. 1949; 5: 296–305.

[39] Goldsmith EW, Renshaw B, Clement CJ, Himschoot EA, Hundertmark KJ, et al Population Structure of Two Rabies Hosts Relative to the Known Distribution of Rabies Virus Variants in Alaska. Mol. Ecol. 2016; 25:675–688. 10.1111/mec.13509 26661691 PMC4738172

[40] MacInnes CD, Smith SM, Tinline RR, Ayers NR, Bachmann P, et al Elimination of rabies from red foxes in eastern Ontario. J Wildl Dis. 2001; 37: 119–132. 11272485 10.7589/0090-3558-37.1.119

[41] Rosatte RC, Power MJ, Donovan D, Davies JC, Allan M, et al Elimination of arctic variant rabies in red foxes, metropolitan Toronto. Emerg Infect Dis. 2007; 13: 25–27. 17370512 10.3201/eid1301.060622 PMC2725809

[42] Johnson N, McElhinney LM, Smith J, Lowings P, Fooks AR. Phylogenetic comparison of the genus Lyssavirus using distal coding sequences of the glycoprotein and nucleoprotein genes. Arch Virol. 2002; 147: 2111–2123. 12417947 10.1007/s00705-002-0877-4

[43] Wu X, Franka R, Velasco-Villa A, Rupprecht CE. Are all lyssavirus genes equal for phylogenetic analyses? Virus Res. 2007; 129: 91–103. 17681631 10.1016/j.virusres.2007.06.022

[44] Aubry KB, Statham MJ, Sacks BN, Perrine JD, Wisely SM. Phylogeography of the North American red fox: vicariance in Pleistocene forest refugia. Mol Ecol. 2009; 18: 2668–2686. 10.1111/j.1365-294X.2009.04222.x 19457180

[45] Geffen E, Waidyaratne S, Dalen L, Angerbjorn A, Vila C, et al Sea ice occurrence predicts genetic isolation in the Arctic fox. Mol Ecol. 2007; 16: 4241–4255. 17868292 10.1111/j.1365-294X.2007.03507.x

[46] Noren K, Angerbjorn A, Hersteinsson P. Population structure in an isolated Arctic fox, Vulpes lagopus, population: the impact of geographical barriers. Biol J Linn Soc. 2009; 97: 18–26.

[47] Bardeleben C, Moore RL, Wayne RK. A molecular phylogeny of the Canidae based on six nuclear loci. Mol Phylogenet Evol. 2005; 37: 815–831. 16213754 10.1016/j.ympev.2005.07.019

[48] Cohen TM, King R, Dolev A, Boldo A, Lichter-Peled A, et al Genetic characterization of populations of the golden jackal and the red fox in Israel. Conserv Genet. 2013; 14: 55–63.

[49] Dalen L, Kvaloy K, Linnell JDC, Elmhagen B, Strand O, et al Population structure in a critically endangered arctic fox population: does genetics matter? Mol Ecol. 2006; 15: 2809–2819. 16911202 10.1111/j.1365-294X.2006.02983.x

[50] Sacks BN, Louie S. Using the dog genome to find single nucleotide polymorphisms in red foxes and other distantly related members of the Canidae. Mol Ecol Resour. 2008; 8: 35–49. 10.1111/j.1471-8286.2007.01830.x 21585716

[51] Zink RM, Barrowclough GF. Mitochondrial DNA under siege in avian phylogeography. Mol Ecol. 2008; 17: 2107–2121. 10.1111/j.1365-294X.2008.03737.x 18397219

[52] Kumar V, Kutschera VE, Nilsson MA, Janke A. Genetic signatures of adaptation revealed from transcriptome sequencing of Arctic and red foxes. BMC Genomics. 2015; 16: 585 10.1186/s12864-015-1724-9 26250829 PMC4528681

[53] Brunker K, Hampson K, Horton DL, Biek R. Integrating the landscape epidemiology and genetics of RNA viruses: rabies in domestic dogs as a model. Parasitology. 2012; 139: 1899–1913. 10.1017/S003118201200090X 22814380 PMC3526958

[54] Mellows A, Barnett R, Dalen L, Sandoval-Castellanos E, Linderholm A, et al The impact of past climate change on genetic variation and population connectivity in the Icelandic arctic fox. Proc R Soc Lond B—Biol Sci. 2012; 279: 4568–4573. 10.1098/rspb.2012.1796 PMC3479732 22977155

[55] Freuling C, Vos A, Johnson N, Mühle RU, Müller T. Rabies In: Johnson N, editor. The role of animals in emerging viral diseases. 1st ed. San Diego: Academic Press 2013; pp. 63–87.

[56] Follmann EH, Ritter DG, Baer GM. Oral rabies vaccination of arctic foxes (Alopex lagopus) with an attenuated vaccine. Vaccine. 1992; 10: 305–308. 1574916 10.1016/0264-410x(92)90368-t

[57] Follmann EH, Ritter DG, Hartbauer DW. Oral Vaccination of Captive Arctic Foxes with Lyophilized SAG2 Rabies Vaccine. J Wildl Dis. 2004; 40: 328–334. 15362836 10.7589/0090-3558-40.2.328

[58] Johnston DH, Fong DW. Epidemiology of arctic fox rabies In: Bögel K, Meslin F-X, Kaplan MM, editors. Wildlife rabies control. Kent, UK: Wells Medical Ltd 1992; pp. 45–49.

[59] Hansen EH. Oral vaccination of arctic foxes in Greenland: A field trial. 7th Annual International Meeting: Advances Towards Rabies Control in the Americas. Atlanta, USA: CDC; 1996. pp. 7.

[60] Kuzmin IV, Botvinkin AD, McElhinney LM, Smith JS, Orciari LA, et al Molecular epidemiology of terrestrial rabies in the former Soviet Union. J Wildl Dis. 2004; 40: 617–631. 15650080 10.7589/0090-3558-40.4.617

[61] Park YJ, Shin MK, Kwon HM. Genetic characterization of rabies virus isolates in Korea. Virus Genes. 2005; 30: 341–347. 15830152 10.1007/s11262-005-6777-4

[62] Nadin-Davis SA, Casey GA, Wandeler A. Identification of regional variants of the rabies virus within the Canadian province of Ontario. J Gen Virol. 1993; 74: 829–837. 8492088 10.1099/0022-1317-74-5-829

[63] Nadin-Davis SA, Casey GA, Wandeler AI. A molecular epidemiological study of rabies virus in central Ontario and western Quebec. J Gen Virol. 1994; 75: 2575–2583. 7931145 10.1099/0022-1317-75-10-2575

[64] Wu H, Wang L, Tao X, Li H, Rayner S, et al Genetic diversity and molecular evolution of the rabies virus matrix protein gene in China. Infect Genet Evol. 2013; 16: 248–253. 10.1016/j.meegid.2013.02.009 23453987

[65] Bourhy H, Reynes JM, Dunham EJ, Dacheux L, Larrous F, et al The origin and phylogeography of dog rabies virus. J Gen Virol. 2008; 89: 2673–2681. 10.1099/vir.0.2008/003913-0 18931062 PMC3326349

[66] Holmes EC, Woelk CH, Kassis R, Bourhy H. Genetic constraints and the adaptive evolution of rabies virus in nature. Virology. 2008; 292: 247–257. 10.1006/viro.2001.1271 11878928

