# Supplementary material for: Spatio-temporal Analysis of the Genetic Diversity of Arctic Rabies Viruses and Their Reservoir Hosts in Greenland
Source: PLoS Negl Trop Dis. 2016 Jul 26;10(7):e0004779. doi: 10.1371/journal.pntd.0004779 (PMC4961414; doi:10.1371/journal.pntd.0004779)
Supplement: S1 Table — (PDF) [file pntd.0004779.s001.pdf]

Supplemental Table S 1: Overview of raw sequence data using NGS generated in this study.

Sample name for this study	Reads	Virus reads	Virus (%)	Mitochondrial Arctic fox reads	Arctic fox mitochondrion (%)
Gra21.05-GRL-1-AF-2005	2,261,322	8,116	0.359	9,145	0.40
Gra07.06-GRL-1-AF-2006	3,175,864	4,451	0.140	4,080	0.13
Gra14.06-GRL-1-AF-2006	3,070,940	1,742	0.057	5,034	0.16
Gra03.13-GRL-1-AF-2013	1,135,200	26,553	2.339	12,181	1.07
Gra23.06-GRL-2-AF-2006	4,375,340	6,120	0.140	4,689	0.11
Gra03.10-GRL-2-AF-2010	3,014,708	6,298	0.209	18,968	0.63
Gra01.13-GRL-2-AF-2013	15,665,420	161,907	1.034	420,692	2.69
Gra02.13-GRL-2-AF-2013	2,086,986	31,788	1.523	16,580	0.79
Gra03.06-GRL-3-AF-2006	3,152,834	4,017	0.127	14,504	0.46
Gra04.06-GRL-3-AF-2006	3,671,700	3,353	0.091	14,724	0.40
Gra05.06-GRL-3-AF-2006	5,093,346	5,548	0.109	7,431	0.15
Gra08.06-GRL-3-AF-2006	4,786,360	10,937	0.229	12,329	0.26
Gra09.06-GRL-3-AF-2006	3,249,052	1,650	0.051	37,730	1.16
Gra10.06-GRL-3-AF-2006	3,315,214	7,961	0.240	15,335	0.46
Gra24.06-GRL-3-D-2006	2,514,468	12,843	0.511	-	-
Gra25.06-GRL-3-AF-2006	4,678,954	6,647	0.142	26,146	0.56
Gra02.07-GRL-3-AF-2007	4,306,408	1,271	0.030	25,190	0.58
Gra01.14-GRL-3-AF-2014	558,922	6,376	1.141	4,115	0.74
Gra16.06-GRL-4-AF-2006	2,984,986	4,391	0.147	5,017	0.17
Gra09.07-GRL-4-AF-2007	3,756,534	8,024	0.214	18,392	0.49
Gra10.07-GRL-4-AF-2007	2,373,734	6,003	0.253	14,557	0.61
Gra02.14-GRL-4-AF-2014	659,242	18,238	2.767	8,380	1.27
Gra03.14-GRL-4-AF-2014	252,966	7,833	3.096	5,137	2.03
Gra02.10-GRL-5-AF-2010	990,010	1,874	0.189	10,234	1.03
Gra05.10-GRL-5-AF-2010	3,525,344	3,449	0.098	21,169	0.60

Gra10.08-GRL-6-AF-2008	2,813,148	9,285	0.330	26,224	0.93
Gra07.09-GRL-6-AF-2009	1,849,544	7,893	0.427	13,411	0.73
Gra13.09-GRL-6-AF-2009	1,767,008	6,464	0.366	8,756	0.50
Gra06.10-GRL-6-AF-2010	4,017,164	1,300	0.032	14,909	0.37
Gra07.10-GRL-6-AF-2010	782,204	1,731	0.221	5,872	0.75
Gra18.05-GRL-7-AF-2005	6,127,458	4,907	0.080	23,915	0.39
Gra01.06-GRL-7-AF-2006	2,847,640	9,704	0.341	11,128	0.39
Gra02.06-GRL-7-AF-2006	1,820,960	6,083	0.334	3,774	0.21
Gra06.06-GRL-7-C-2006	2,235,074	2,075	0.093	-	-
Gra20.06-GRL-7-AF-2006	584,502	352	0.060	446	0.08
Gra21.06-GRL-7-AF-2006	9,149,950	841	0.009	43,490	0.48
Gra26.06-GRL-7-AF-2006	356,468	138	0.039	635	0.18
Gra04.07-GRL-7-AF-2007	4,803,878	27,032	0.563	41,071	0.85
Gra05.07-GRL-7-AF-2007	2,611,946	1,668	0.064	26,106	1.00
Gra15.07-GRL-7-S-2007	4,345,878	27,895	0.642	-	-
Gra3a.07-GRL-7-AF-2007	3,834,316	4,778	0.125	18,062	0.47
Gra3b.07-GRL-7-AF-2007	3,436,546	2,076	0.060	18,834	0.55
Gra02.08-GRL-7-AF-2008	2,784,906	724	0.026	4,597	0.17
Gra03.08-GRL-7-AF-2008	2,278,708	2,302	0.101	22,591	0.99
Gra13.08-GRL-7-AF-2008	3,435,630	13,751	0.400	25,182	0.73
Gra01.09-GRL-7-S-2009	4,996,346	4,558	0.091	-	-
Gra02.09-GRL-7-S-2009	2,603,664	7,220	0.277	-	-
Gra03.09-GRL-7-AF-2009	2,750,960	620	0.023	2,273	0.08
Gra04.09-GRL-7-AF-2009	2,917,080	1,055	0.036	24,280	0.83
Gra01.10-GRL-7-AF-2010	2,953,802	2,331	0.079	13,201	0.45
Gra01.11-GRL-7-AF-2011	4,915,266	8,072	0.164	-	-
13232-CAN-NT-AF-1977	1,129,456	8,467	0.750	10,078	0.89

RV1391-GRL-1-F-1990	6,378,046	15,921	0.250	-	-
RV1415-GRL-4-F-2001	1,952,228	1,248	0.064	-	-
RV1416-GRL-5-AF-2002	4,012,672	60,932	1.518	21,314	0.53
RV1417-GRL-3-AF-2002	4,429,870	77,781	1.756	4,702	0.11
RV1418-GRL-4-AF-2002	4,044,772	51,947	1.284	14,161	0.35
RV1419-GRL-3-AF-2002	4,845,248	23,611	0.487	4,418	0.09
RV1420-GRL-4-AF-2002	7,331,438	30,013	0.409	3,327	0,05
