# Supplementary material for: Spatio-temporal Analysis of the Genetic Diversity of Arctic Rabies Viruses and Their Reservoir Hosts in Greenland
Source: PLoS Negl Trop Dis. 2016 Jul 26;10(7):e0004779. doi: 10.1371/journal.pntd.0004779 (PMC4961414; doi:10.1371/journal.pntd.0004779)
Supplement: S2 Table — (PDF) [file pntd.0004779.s002.pdf]

Supplemental Table S 1: Reference sequences used in supplement Figure 1.

Name	Accession	Year	Country	Host	Region	Sequence length	Reference
AY352462 RVHK Russia 1998	AY352462	1998	Russia	Homo sapiens	Krasnoyarsk	1414	[60]
AY352486 3510w Russia 1995	AY352486	1995	Russia	Wolf	Yakutia	1414	[60]
AY352488 743a Russia 1988	AY352488	1988	Russia	Arctic fox	Yakutia	1414	[60]
AY352498 4795 USA 1988	AY352498	1988	USA	Dog	Alaska	1414	[60]
AY352499 1420 USA	AY352499	-	USA	Red fox	Alaska	1414	[60]
AY352500 1421 USA 1988	AY352500	1988	USA	Red fox	Alaska	1414	[60]
AY352501 1422 USA	AY352501	-	USA	Arctic fox	Alaska	1414	[60]
AY352514 RV294 Russia 1990	AY352514	1990	Russia	Arctic fox	Yakutia	405	[60]
DQ010123 RV53 USA	DQ010123	-	USA	Fox	Maine	400	[16]
DQ010124RV163 Canada 1989	DQ010124	1989	Canada	Arctic fox	-	400	[16]
DQ010125 RV255 Russia 1988	DQ010125	1988	Russia	Arctic fox	Yakutia	400	[16]
DQ010126 RV441 Belarus	DQ010126	-	Belarus	Fox	-	400	[16]
DQ010127 RV443 Russia 1990	DQ010127	1990	Russia	Horse	Yakutia	400	[16]
DQ010129 RV1336 Russia 2002	DQ010129	2002	Russia	Arctic fox	Yakutia	400	[16]
DQ010131 RV1338 Russia 2002	DQ010131	2002	Russia	Arctic fox	Yakutia	400	[16]
DQ010132 RV1391 Greenland 1990	DQ010132	1990	Greenland	Arctic fox	Thule	400	[16]
DQ010136 RV1396 Greenland 1991	DQ010136	1991	Greenland	Arctic fox	Sisimiut	400	[16]
DQ010141 RV1407 Greenland 1994	DQ010141	1994	Greenland	Arctic fox	Upernavik	400	[16]
DQ010143 RV1413 Greenland 1997	DQ010143	1997	Greenland	Arctic fox	Gronnedal	400	[16]
DQ010147 RV1419 Greenland 2002	DQ010147	2002	Greenland	Arctic fox	Ilulissat	400	[16]
DQ010148 RV1420 Greenland 2002	DQ010148	2002	Greenland	Arctic fox	Kangerlussuaq	400	[16]
EF611831 SG22 Russia 1987	EF611831	1987	Russia	Arctic fox	Yakutia	1350	[17]
EF611833 SG23 Russia 1988	EF611833	1988	Russia	Arctic fox	Yakutia	1350	[17]
EF611842 A6086 USA 2006	EF611842	2006	USA	Red fox	Alaska	1350	[17]
EF611843 A7027 USA 2007	EF611843	2007	USA	Red fox	Alaska	1350	[17]
EF611844 A7006 USA 2007	EF611844	2007	USA	Dog	Alaska	1350	[17]

EF611845 A7033 USA 2007	EF611845	2007	USA	Red fox	Alaska	1350	[17]
EF611849 A6091 USA 2006	EF611849	2006	USA	Red fox	Alaska	1350	[17]
EF611850 A7032 USA 2007	EF611850	2007	USA	Red fox	Alaska	1350	[17]
EF611851 A7031 USA 2006	EF611851	2006	USA	Arctic fox	Alaska	1350	[17]
EF611852 A7026 USA 2006	EF611852	2006	USA	Arctic fox	Alaska	1350	[17]
EF611853 A0905 USA 2006	EF611853	2006	USA	Dog	Alaska	1350	[17]
EF611854 A0904 USA 2006	EF611854	2006	USA	Red fox	Alaska	1350	[17]
EF611855 A0903 USA 2006	EF611855	2006	USA	Dog	Alaska	1350	[17]
EF611856 A0906 USA 2006	EF611856	2006	USA	Arctic fox	Alaska	1350	[17]
JN258416 90V819AL USA 1990	JN258416	1990	USA	Arctic fox	Alaska	500	[18]
JN258417 90V820AL USA 1990	JN258417	1990	USA	Red fox	Alaska	500	[18]
JN258418 90V821AL USA 1990	JN258418	1990	USA	Wolf	Alaska	500	[18]
JN258419 90V822AL USA 1990	JN258419	1990	USA	Red fox	Alaska	500	[18]
JN258420 89V804AL USA 1989	JN258420	1989	USA	Red fox	Alaska	500	[18]
JN258421 89V803AL USA 1989	JN258421	1989	USA	Red fox	Alaska	500	[18]
JN258422 89V812AL USA 1989	JN258422	1989	USA	Red fox	Alaska	500	[18]
JN258423 89V813AL USA 1989	JN258423	1989	USA	Red fox	Alaska	500	[18]
JN258424 90V815AL USA 1990	JN258424	1990	USA	Red fox	Alaska	500	[18]
JN258425 90V816AL USA 1990	JN258425	1990	USA	Arctic fox	Alaska	500	[18]
JN258426 90V818AL USA 1990	JN258426	1990	USA	Arctic fox	Alaska	500	[18]
JN258427 89V805AL USA 1989	JN258427	1989	USA	Dog	Alaska	500	[18]
JN258428 89V806AL USA 1989	JN258428	1989	USA	Red fox	Alaska	500	[18]
JN258429 89V807AL USA 1989	JN258429	1989	USA	Wolf	Alaska	500	[18]
JN258430 89V808AL USA 1989	JN258430	1989	USA	Red fox	Alaska	500	[18]
JN258431 89V809AL USA 1989	JN258431	1989	USA	Arctic fox	Alaska	500	[18]
JN258432 89V810AL USA 1989	JN258432	1989	USA	Dog	Alaska	500	[18]
JN258433 89V811AL USA 1989	JN258433	1989	USA	Red fox	Alaska	500	[18]
JN258434 90L3187CAN Canada 1990	JN258434	1990	Canada	Arctic fox	Northwest Territories	500	[18]
JN258435 90L3310CAN Canada 1990	JN258435	1990	Canada	Arctic fox	Northwest Territories	500	[18]

JN258436 91L0250CAN Canada 1991	JN258436	1991	Canada	Red fox	Northwest Territories	500	[18]
JN258437 93L0459CAN Canada 1993	JN258437	1993	Canada	Arctic fox	Northwest Territories	500	[18]
JN258438 93L0669CAN Canada 1993	JN258438	1993	Canada	Arctic fox	Northwest Territories	500	[18]
JN258439 93L2863CAN Canada 1993	JN258439	1993	Canada	Arctic fox	Northwest Territories	500	[18]
JN258440 95L1947CAN Canada 1995	JN258440	1995	Canada	Fox	Northwest Territories	500	[18]
JN258441 97L1793CAN Canada 1997	JN258441	1997	Canada	Arctic fox	Northwest Territories	500	[18]
JN258442 05L0110CAN Canada 2005	JN258442	2005	Canada	Red fox	Nunavut	500	[18]
JN258443 05N6048CAN Canada 2005	JN258443	2005	Canada	Red fox	Manitoba	500	[18]
JN258444 02N0897CAN Canada 2002	JN258444	2002	Canada	Arctic fox	Nunavut	500	[18]
JN258445 06L0122CAN Canada 2006	JN258445	2006	Canada	Red fox	Northwest Territories	500	[18]
JN258446 05L0250CAN Canada 2005	JN258446	2005	Canada	Dog	Northwest Territories	500	[18]
JN258447 05L0111CAN Canada 2005	JN258447	2005	Canada	Arctic fox	Nunavut	500	[18]
JN258448 07L1475CAN Canada 2007	JN258448	2007	Canada	Arctic fox	Nunavut	500	[18]
JN258449 08L1328CAN Canada 2008	JN258449	2008	Canada	Red fox	Nunavut	500	[18]
JN258450 08L0047CAN Canada 2008	JN258450	2008	Canada	Arctic fox	Nunavut	500	[18]
JN258451 02L0035CAN Canada 2002	JN258451	2002	Canada	Red fox	Nunavut	500	[18]
JN258452 06L0213CAN Canada 2006	JN258452	2006	Canada	Arctic fox	Nunavut	500	[18]
JN258453 99L0142CAN Canada 1999	JN258453	1999	Canada	Arctic fox	Nunavut	500	[18]
JN258454 99L2159CAN Canada 1999	JN258454	1999	Canada	Arctic fox	Nunavut	500	[18]
JN258455 07L0005CAN Canada 2007	JN258455	2007	Canada	Arctic fox	Northwest Territories	500	[18]
JN258456 91L0085CAN Canada 1991	JN258456	1991	Canada	Arctic fox	Northwest Territories	500	[18]
JN258457 92L0195CAN Canada 1992	JN258457	1992	Canada	Fox	Northwest Territories	500	[18]
JN258458 92L0084CAN Canada 1992	JN258458	1992	Canada	Fox	Northwest Territories	500	[18]
JN258459 93L0286CAN Canada 1993	JN258459	1993	Canada	Arctic fox	Northwest Territories	500	[18]
JN258460 91L0252CAN Canada 1991	JN258460	1991	Canada	Arctic fox	Northwest Territories	500	[18]
JN258461 92L0228CAN Canada 1992	JN258461	1992	Canada	Arctic fox	Northwest Territories	500	[18]
JN258462 90L3225CAN Canada 1990	JN258462	1990	Canada	Arctic fox	Northwest Territories	500	[18]
JN258463 91L0165CAN Canada 1991	JN258463	1991	Canada	Arctic fox	Northwest Territories	500	[18]
JN258464 93L2980CAN Canada 1993	JN258464	1993	Canada	Arctic fox	Northwest Territories	500	[18]

JN258465 93N2764CAN Canada 1993	JN258465	1993	Canada	Arctic fox	Québec	500	[18]
JN258466 08L1453CAN Canada 2008	JN258466	2008	Canada	Arctic fox	Northwest Territories	500	[18]
JN258467 93N0782CAN Canada 1993	JN258467	1993	Canada	Arctic fox	Northwest Territories	500	[18]
JN258468 94N3803CAN Canada 1994	JN258468	1994	Canada	Arctic fox	Northwest Territories	500	[18]
JN258469 04L1518CAN Canada 2004	JN258469	2004	Canada	Arctic fox	Nunavut	500	[18]
JN258470 07N0248CAN Canada 2007	JN258470	2007	Canada	Arctic fox	Nunavut	500	[18]
JN258471 98L1893CAN Canada 1998	JN258471	1998	Canada	Arctic fox	Northwest Territories	500	[18]
JN258472 98L2209CAN Canada 1998	JN258472	1998	Canada	Arctic fox	Northwest Territories	500	[18]
JN258473 95N1082CAN Canada 1995	JN258473	1995	Canada	Arctic fox	Ontario	500	[18]
JN258474 99N0549CAN Canada 1999	JN258474	1999	Canada	Arctic fox	Québec	500	[18]
JN258475 07L0298CAN Canada 2007	JN258475	2007	Canada	Arctic fox	Nunavut	500	[18]
JN258476 97L0270CAN Canada 1997	JN258476	1997	Canada	Red fox	Northwest Territories	500	[18]
JN258477 92L0491CAN Canada 1992	JN258477	1992	Canada	Arctic fox	Northwest Territories	500	[18]
JN258478 06N5386CAN Canada 2006	JN258478	2006	Canada	Red fox	Nunavut	500	[18]
JN258479 01N12568CAN Canada 2001	JN258479	2001	Canada	Arctic fox	Northwest Territories	500	[18]
JN258480 91N5406CAN Canada 1991	JN258480	1991	Canada	Wolf	Ontario	500	[18]
JN258481 93N0398CAN Canada 1993	JN258481	1993	Canada	Arctic fox	Northwest Territories	500	[18]
JN258482 96N1804CAN Canada 1996	JN258482	1996	Canada	Arctic fox	Québec	500	[18]
JN258483 96N1855CAN Canada 1996	JN258483	1996	Canada	Red fox	Newfoundland	500	[18]
JN258484 96N2026CAN Canada 1996	JN258484	1996	Canada	Red fox	Newfoundland	500	[18]
JN258485 96N2393CAN Canada 1996	JN258485	1996	Canada	Red fox	Newfoundland	500	[18]
JN258486 96N2397CAN Canada 1996	JN258486	1996	Canada	Otter	Québec	500	[18]
JN258487 02N2909CAN Canada 2002	JN258487	2002	Canada	Red fox	Newfoundland	500	[18]
JN258488 02N3548CAN Canada 2002	JN258488	2002	Canada	Red fox	Newfoundland	500	[18]
JN258489 03N3362CAN Canada 2003	JN258489	2003	Canada	Red fox	Newfoundland	500	[18]
JN258490 00L0417CAN Canada 2000	JN258490	2000	Canada	Red fox	Northwest Territories	500	[18]
JN258491 00L0528CAN Canada 2000	JN258491	2000	Canada	Red fox	Northwest Territories	500	[18]
JN258492 00N1512CAN Canada 2000	JN258492	2000	Canada	Red fox	Ontario	500	[18]
JN258493 00N2483CAN Canada 2000	JN258493	2000	Canada	Dog	Québec	500	[18]

JN258494 00N3420CAN Canada 2000	JN258494	2000	Canada	Dog	Québec	500	[18]
JN258495 01N1650CAN Canada 2001	JN258495	2001	Canada	Red fox	Newfoundland	500	[18]
JN258496 01N3447CAN Canada 2001	JN258496	2001	Canada	Red fox	Québec	500	[18]
JN258497 00L0408CAN Canada 2000	JN258497	2000	Canada	Red fox	Ontario	500	[18]
JN258498 03N4056CAN Canada 2003	JN258498	2003	Canada	Red fox	Newfoundland	500	[18]
JN258499 09N0671CAN Canada 2009	JN258499	2009	Canada	Red fox	Québec	500	[18]
JN258500 90L0043CAN Canada 1990	JN258500	1990	Canada	Bear	Northwest Territories	500	[18]
JN258501 90V865GRN Greenland 1990	JN258501	1990	Greenland	-	-	500	[18]
JN258502 91V867GRN Greenland 1991	JN258502	1991	Greenland	Fox	Scoresbysund	500	[18]
JN258503 92V868GRN Greenland 1992	JN258503	1992	Greenland	Fox	Moriussiaq	500	[18]
JN258504 92V869GRN Greenland 1992	JN258504	1992	Greenland	Fox	Holsteinsborg	500	[18]
JN258505 92V870GRN Greenland 1992	JN258505	1992	Greenland	-	-	500	[18]
JN258506 93V871GRN Greenland 1993	JN258506	1993	Greenland	Dog	Holsteinsborg	500	[18]
JN258507 93V872GRN Greenland 1993	JN258507	1993	Greenland	-	-	500	[18]
JN258508 94V873GRN Greenland 1994	JN258508	1994	Greenland	Sheep	Julianehab	500	[18]
JN258509 94V876GRN Greenland 1994	JN258509	1994	Greenland	-	-	500	[18]
JN258510 95V880GRN Greenland 1995	JN258510	1995	Greenland	Fox	Station Nord	500	[18]
JN258511 97V881GRN Greenland 1997	JN258511	1997	Greenland	Sheep	Julianehab	500	[18]
JN258512 00V882GRN Greenland 2000	JN258512	2000	Greenland	Fox	Frederikshab	500	[18]
JN258513 00V883GRN Greenland 2000	JN258513	2000	Greenland	Fox	Christianshab	500	[18]
JN258514 01V884GRN Greenland 2001	JN258514	2001	Greenland	Fox	Nanortalik	500	[18]
JN258515 01V886GRN Greenland 2001	JN258515	2001	Greenland	Fox	Sukkertoppen	500	[18]
JN258516 01V887GRN Greenland 2001	JN258516	2001	Greenland	Fox	Christianshab	500	[18]
JN258517 01V888GRN Greenland 2001	JN258517	2001	Greenland	Fox	Nuuk	500	[18]
JN258518 01V891GRN Greenland 2001	JN258518	2001	Greenland	-	-	440	[18]
JN258519 01V893GRN Greenland 2001	JN258519	2001	Greenland	Fox	-	500	[18]
JN258520 02V895GRN Greenland 2002	JN258520	2002	Greenland	Fox	Nuuk	500	[18]
JN258521 02V897GRN Greenland 2002	JN258521	2002	Greenland	Fox	Kangerlussuaq	500	[18]
JN258522 02V898GRN Greenland 2002	JN258522	2002	Greenland	Fox	Egedesminde	500	[18]

JN258523 02V899GRN Greenland 2002	JN258523	2002	Greenland	Fox	Thule	500	[18]
JN258586 07V1479AL USA 2007	JN258586	2007	USA	Red fox	Alaska	1360	[18]
JN258587 07V1480AL USA 2007	JN258587	2007	USA	Red fox	Alaska	1360	[18]
JN258588 07V1481AL USA 2007	JN258588	2007	USA	Arctic fox	Alaska	1360	[18]
JN258589 08V1484AL USA 2008	JN258589	2008	USA	Red fox	Alaska	1360	[18]
JN258590 08V1485AL USA 2008	JN258590	2008	USA	Red fox	Alaska	1360	[18]
JN258591 08V1486AL USA 2008	JN258591	2008	USA	Red fox	Alaska	1360	[18]
JN258592 08V1488AL USA 2008	JN258592	2008	USA	Arctic fox	Alaska	1360	[18]
L20673 91RABN1578 Canada 1991	L20673	1991	Canada	Arctic fox	Ontario	1365	[62]
L20674 91RABN2756 Canada 1991	L20674	1991	Canada	Arctic fox	Ontario	1365	[62]
L20675 91RABN0783 Canada 1991	L20675	1991	Canada	Arctic fox	Ontario	1365	[62]
L20676 90RABN9196 Canada 1990	L20676	1990	Canada	Arctic fox	Ontario	1365	[62]
U03768 Ontario Type 5 - 8480FX Canada 1993	U03768	1993	Canada	Red fox	Ontario	1367	[63]
U03769 Arctic A1 - 1090DG Canada 1993	U03769	1993	Canada	Dog	-	1367	[63]
U03770 Hudson Bay - 4055DG Canada 1992	U03770	1992	Canada	Dog	-	1367	[63]
U11734 1991 fox 6199 Canada 1991	U11734	1991	Canada	Red fox	Ontario	1455	(Nadin-Davis et al., unpublished)
U11735 1993 fox 2244 Canada 1993	U11735	1993	Canada	Red fox	Ontario	1455	(Nadin-Davis et al., unpublished)
U22654 8684GRO Greenland 1981	U22654	1981	Greenland	Arctic fox	-	1350	[15]
U22655 9105CAN Canada 1990	U22655	1990	Canada	Red fox	Ontario	1350	[15]
